# C3G Downregulation Enhances Stemness in Glioblastoma Cells by Promoting PKM2 Upregulation

**DOI:** 10.7150/ijbs.126349

**Published:** 2026-07-01

**Authors:** Mateo Cueto-Remacha, Sara Manzano, Minerva Iniesta-González, Jaime Mancebo, Paula Martin-Serna, Nerea Palao, Cristina Baquero, Andrea R. López-Pastor, Cristina Peralbo-Avilés, Alvaro Gutierrez-Uzquiza, Ángel M Cuesta, Paloma Bragado, Carmen Guerrero, Almudena Porras

**Affiliations:** 1 Departamento de Bioquímica y Biología Molecular, Facultad de Farmacia, Universidad Complutense de Madrid, 28040 Madrid, Spain.; 2 Instituto de Investigación Sanitaria del Hospital Clínico San Carlos (IdISSC), 28040 Madrid, Spain.; 3 Laboratorio de Genética y bases moleculares de enfermedades complejas. Instituto de Investigación Sanitaria del Hospital Clínico San Carlos (IdISSC), 28040 Madrid, Spain.; 4 Redes de Investigación Cooperativa de resultados en salud, RICORS, REI (Red de enfermedades inflamatorias); 5 Centro de Investigación del Cáncer, Universidad de Salamanca-CSIC, 37007 Salamanca, Spain.; 6 Instituto de Investigación Biomédica de Salamanca (IBSAL), 37007 Salamanca, Spain.; 7 Departamento de Medicina, Universidad de Salamanca, 37007 Salamanca, Spain.

**Keywords:** C3G/RapGEF1, glioblastoma, PKM2, stemness

## Abstract

Glioblastoma (GBM), the most common and aggressive primary brain tumor, exhibits profound metabolic reprograming that sustains its progression and therapy resistance. Our previously published work demonstrated that C3G expression is downregulated in GBM, which enhances migration and invasion. Here, we show that C3G silencing or knockout in GBM cells reprograms glucose metabolism favoring glycolysis and lactate production through upregulation of PKM2 and LDHA. Furthermore, Seahorse metabolic profiling further revealed increased respiratory capacity and glycolysis upon C3G downregulation or depletion. Mechanistically, C3G silencing increases the levels of the splicing factor PTBP1, which forces *PKM* splicing towards PKM2 expression as demonstrated by transient PTBP1 silencing, although other splicing factors such as SRSF3 could also contribute to PKM2 expression. Additionally, C3G downregulation or depletion enhances sphere formation, stemness and tumor initiating capacity in GBM cells, which is rescued by C3G re-expression in C3G knockout GBM cells. This enhanced stemness induced by C3G silencing in GBM cells is prevented by transient PTBP1 or PKM2 silencing or pharmacological inhibition of PKM2 with compound 3K, which also decreases cell viability within the spheres (3D-cell cultures) and the expression of stemness markers. However, in 2D-cell cultures compound 3K increases cell viability in C3G-silenced GBM cells, while decreasing that of C3G knockout cells. This supports a specific role for C3G/PKM2 axis in GBM cancer stem cells through mechanisms likely dependent on both PKM2-mediated metabolic reprogramming and nuclear effects on gene transcription. Altogether, our findings identify C3G as a novel regulator of GBM metabolism and stemness acting through PKM2, unveiling new potential diagnostic and therapeutic implications for C3G in a subset of GBM patients.

## Introduction

Glioblastoma (GBM) is a highly malignant grade IV glioma 1, which is considered the most aggressive primary brain tumor 2,3 due to its high recurrence rate and treatment resistance 4,5. GBM tumors are characterized by high intra- and inter-GBM tumor heterogeneity and the presence of GBM stem cells (GSCs) 6,7,8. GSCs are a subpopulation of cells with self-renewal and differentiation capacities, which contribute to tumor initiation, progression and recurrence due to treatment resistance 5,9. Hence, some new therapies are focused on targeting GSCs.

GBMs have been classified in different ways by World Health Organization (WHO) according to genetic alterations (i. e. IDH (isocitrate dehydrogenase) mutant type) and histological characteristics 10. However, these classifications failed to predict prognosis and response to treatment. Hence, another classification has also considered biological activities, defining four functional subtypes: 1) proliferative/progenitors or 2) neurons, belonging to the neurodevelopment branch, and 3) mitochondrial (MTC) or 4) glycolytic/plurimetabolic (GPM), included in the metabolic branch 11. MTC tumors are dependent on oxidative phosphorylation (OXPHOS) and are associated with better outcome due to the efficacy of antimetabolic treatments, while GPM cells are more resistant to treatments.

GBM metabolic rewiring is crucial for tumor growth, progression and resistance to treatment 12. Glycolysis, lactate production and lipid metabolism are upregulated in GBM to maintain cell survival, directly and through regulation of the tumor microenvironment 12. Specifically, glycolysis and the Warburg effect play a pivotal role in GBM, favoring GBM tumor growth and progression 13,14. Stemness is also reinforced by enhanced glycolysis, with an established association between stem cell-like GBM tumor subpopulations, elevated basal glycolytic activity and increased expression of glycolytic enzymes such as PKM2 6,15. Hence, glycolysis represents the main source of energy for specific populations of GSCs 6. Moreover, PKM2 inhibition has been reported to inhibit proliferation, migration and invasion of GBM cells 16,17, while an increase in PKM2 activity by inducing the formation of a tetramer exerts opposite effects in some contexts 18. In this line, it was described that PKM2 promoted self-renewal and chemoresistance in GBM 19.

C3G (RAPGEF1) is a guanine nucleotide exchange factor (GEF) for Rap1 and other members of Ras superfamily 20,21, which also act through GEF independent mechanisms 22,23,24. C3G regulates several cellular functions such as proliferation, differentiation, apoptosis, adhesion and migration 21,24,25,26,27,28,29,30,31,32. In cancer, the role of C3G depends on the tumor type and stage 29,33. High expression of p87C3G isoform has been associated with chronic myeloid leukemia development 34. C3G is also upregulated in hepatocarcinoma (HCC), inducing tumor growth, while decreasing invasion 35. Similarly, in colorectal cancer (CRC), C3G plays a dual role 29. In highly invasive breast carcinoma cells, C3G reduces migration 36. In GBM, we have previously demonstrated that C3G expression is downregulated in patient-derived GBM samples and GBM cells compared with astrocytes. Moreover, an additional decrease in C3G through gene silencing promotes an epithelial-mesenchymal transition-like process in GBM cells that enhances their migration and invasion. Besides, the activation of several receptor tyrosine kinases (RTKs) such as FGFR1 is enhanced, which promotes invasion in C3G-silenced GBM cells. C3G downregulation also increases tumor volume in *in vivo* assays, even though cell density within tumors is reduced 37.

Based on this key role of C3G in GBM progression and the relevance of metabolism reprogramming enhancing glycolysis in GBM 14, as well as the function of C3G 38 and its target, Rap1, in regulating liver glucose metabolism 39, we hypothesized that C3G could also rewire GBM metabolism. Additionally, in view of the correlation between low levels of C3G and GBM tumor progression in patients 37, C3G down-regulation could also increase GBM cells' stemness capacity.

## Material and Methods

### Cell culture and treatments

The human GBM U87MG (abbreviated as U87) (from ATCC, HTB-14) and the non-commercial GBM 12Ф12 cell line with a stem-like phenotype, derived from a patient GBM tumor 40 (mycoplasma free), were used. C3G-silenced U87 and 12Ф12 cells were previously generated in the laboratory using lentiviral particles with shRNAs against human C3G 37 and U87C3GKO cells using CRISPR-Cas9 technology. C3G-silenced cells were selected with puromycin 1 μg/mL (Panreac Applichem A2856) and cells transduced with Cas9 were selected with blasticidine 10 μg/mL (Sigma 15205).

Cells were grown in DMEM supplemented with 10% fetal bovine serum (FBS) under attachment conditions for 2D assays.

To inhibit PKM2, cells were treated with the PKM2 inhibitor compound 3K (SelleckChem S8616) prepared as a 10 mM solution in DMSO.

### Generation of spheres

GBM neurospheres were generated by maintaining U87 and 12Ф12 cells in DMEM:Ham's F-12 medium (1:1 mixture; Corning 10-013-CV, Corning 10-080-CV) supplemented with 1% penicillin/streptomycin (Sigma P3032, Sigma 13752), 20 mM HEPES (ThermoScientific J60712.AP), 2 mM glutamine (Lonza H3BEBP17-605E), 1% B27 (Gibco 17504044), 20 ng/mL EGF (Corning 354052), 20 ng/mL FGF2 (Gibco PHG0026), 2 μg/mL sodium heparin (Sigma Aldrich H3149-25KU), and 1 μg/mL leukemia inhibitory factor (Millipore LIF1010), using non-adherent dishes (Corning 430589). Cells were incubated at 37ºC in a humified atmosphere with 5% CO_2_ for 7-10 days, until neurospheres reached ~150 μm in diameter, at which point neurospheres were passaged. Neurospheres were dissociated using Accutase™ (Corning 25058CI) and replated as single cells in new non-adherent dishes.

Spheres were subcultured for 1-9 passages, and representative images were acquired at each passage using Eclipse TE300 microscope (Nikon).

### Western blot analysis

Protein extracts and western blot analysis were carried out as described 37. Membranes were probed with primary antibodies at 1:1000 dilution or as indicated: C3G (Bethyl A301-965A), PKM2 (Cell signaling 3198S, 1:10000), PKM1 (Cell signaling 7067S), LDHA (Cell signaling 2012S, 1:5000), PTBP1 (Cell signaling 57246S, 1:5000), SRSF3 (SelleckChem F3211), Nestin (Abcam ab105389, 1:500), GFAP (Dako Z033429-2, 1:2500), SOX2 (Cell signaling 3579, 1:500), β-actin (Cell signaling 3700, 1:2500) and α-Tubulin (Cell signaling 3873S, 1:2500).

### RNA extraction and RT-qPCR analysis

Total RNA isolation and RT-qPCR analysis was performed as described 37. Total RNA was isolated using NucleoSpin RNA kit (Macherey-Nagel 740955.50) and reverse transcribed using SuperScript IV RT kit (Invitrogen 18090200). cDNA was amplified using specific primers for *PTBP1*, *PKM1*, *PKM2*, *SRSF2*, *SRSF3*, *hnRNPA1*, *hnRNPA2/B1*, *POU5F1*, *SOX2*, *NANOG*, *BMI1*, *PROM1*, *CD44* and *TBP* (see Supplementary Information for sequences) to normalize, and detected by SYBR Green (Roche 04913850001) using Thermo Fisher Scientific Applied Biosystems QuantStudio 5 Real-Time PCR System. Ct (threshold cycle) for a gene minus Ct for TBP=ΔCt and then, referred to non-silenced control values (sample ΔCt-non-silenced ΔCt= ΔΔCt) to calculate RQ (2^-ΔΔCt^).

### Wide proteomic analysis

Proteome-wide analysis was performed at the Proteomics Scientific and Technical Service of IACS (Zaragoza, Spain). U87 parental and C3G-silenced cells were grown to 80% confluence and serum-starved for 24 h prior to lysis with 6 M urea/0.1% SDS. Lysates were sonicated, clarified by centrifugation, and protein concentration was determined by the Lowry method. Differential protein expression between conditions was analyzed by two-dimensional differential in-gel electrophoresis (2D-DIGE) (n=4). Protein spots of interest were excised, digested with trypsin, and identified by MALDI-TOF/TOF and LC-ESI-MS/MS. Only when a minimum number of peptides corresponding to the same protein showed consistent increases or decreases across the 4 biological replicates was it considered that this protein changed. Bioinformatic analyses of differentially expressed proteins were performed using Panther, Cancertool, and DAVID (accessed December 2019) to assess enrichment in GO biological processes (GOBP), cellular components (GOCC), molecular functions (GOMF), Reactome pathways, and KEGG pathways under default parameters.

### Targeted metabolomic analysis

A targeted metabolomic analysis of metabolites from the trycarboxylic acid (TCA) cycle was performed using the metabolomics platform at CIC bioGUNE (Derio, Spain), The analysis included a quantification of glucose, fumarate, citrate, malate, and succinate. Detection, quantification, and identification of the metabolites were carried out by ultra-performance liquid chromatography (UPLC) coupled to time-of-flight mass spectrometry (ToF MS, SYNAPT G2 HDMS).

### Analysis of metabolic activity using Seahorse

Metabolic activity of C3G-silenced U87 and 12Ф12 cells, as well as U87C3GKO cells and their corresponding controls, was assessed using a Seahorse XFp extracellular flux analyzer (Agilent, Santa Clara, CA, USA). Oxygen consumption rate (OCR) and extracellular acidification rate (ECAR) were simultaneously measured in 1.2 × 10^5^ U87 cells or 1 × 10^5^ 12Ф12 cells seeded onto XFp cell culture miniplates (Agilent 103022-100) one day prior to the assay. On the day of analysis, culture medium was replaced with XF DMEM medium, pH 7.4 (Agilent 103575-100), supplemented with glucose (10 mM; Santa Cruz Biotechnology sc-221678), glutamine (Lonza BEBP17-605E) and pyruvate (2 mM; Santa Cruz Biotechnology sc-286966). Each condition was analyzed in triplicate with a Cell Mito Stress Test Kit (Agilent 103010-100), following the manufacturer's instructions. Briefly, OCR and ECAR were measured in basal conditions and after sequential injections of oligomycin (1.5 μM), carbonylcyanide-p-trifluoromethoxyphenylhydrazone (FCCP, 0.5 μM), and a mixture of rotenone and antimycin A (both at 0.5 μM) injections. Data were normalized to protein content.

### Lactate production

Lactate released by cells was quantified using medium collected from cell cultures at 4, 24, and 48 h, and stored at -80ºC until analysis. A calibration curve of 0-10 mM lactate (Fluka 69775) in serum-free DMEM was prepared in parallel. For each sample, 16 μL of culture medium were mixed with 217 μL of 0.4 M hydrazine (Sigma 225819) -0.5 M glycine (Panreac A1067) buffer and 16 μL of 17 mg/mL NAD^+^ (Sigma 43420) solution. Absorbance at 340 nm was measured before and after addition of 2 U lactate dehydrogenase (Roche 10127876001), followed by incubation for 1 h at 25ºC. The increase in absorbance, reflecting NADH formation, was used to calculate lactate concentration. Lactate levels in DMEM supplemented with 10% FBS were subtracted as background. Results were normalized to total protein content of the cell extracts.

### Immunofluorescence analysis of PKM2

Cells were seeded on glass coverslips pre-coated with 2% gelatin (Sigma G9391) and maintained in culture for 24 h in medium supplemented with 10% FBS. Then, cells were washed twice with PBS, fixed with 4% PFA for 20 min at room temperature (RT), washed with PBS, and permeabilized with PBS containing 0.1% Triton X-100 and 0.5% BSA for 20 min at RT. After permeabilization, cells were incubated with blocking solution (5% BSA, 1.5% goat serum in PBS) for 1 h at RT, followed by overnight incubation at 4ºC with anti-PKM2 antibody (Cell signaling 3198S) diluted 1:100 in blocking solution. Cells were then washed with PBS and incubated with secondary antibody (goat anti-rabbit-Alexa 555; Invitrogen A32732; 1:250) and DAPI (Sigma D9542; 1 μg/mL) in blocking solution. After final PBS washes, coverslips were mounted with mounting medium (Invitrogen P36930). Images were acquired using a Nikon Eclipse TE300 microscope coupled to a camera. Fluorescence intensity was quantified using ImageJ software.

### Orthotopic xenograft assay

Orthotopic xenotransplants were established using parental and shC3G U87 cells. Briefly, 8-week-old athymic female nude Foxn1nu mice (Envigo, Barcelona, Spain) were subjected to stereotactically guided intracranial injections. A total of 2.4 × 10^5^ cells, resuspended in 4 μL of culture medium were injected into the striatum (coordinates: A-P, -0.5 mm; M-L, +2 mm; D-V, -3 mm; related to Bregma) using a Hamilton syringe. Animals were monitored daily and sacrificed at the onset of neurological symptoms. Six weeks post-injection, magnetic resonance imaging (MRI) was performed to determine tumor size, and samples were resected and processed for further analysis.

All animal experiments were carried out in compliance with the European Community Council Directive (2010/63/EU) and following guidelines for animal research from Complutense University Ethical Committee, approved by Comunidad de Madrid (Spain) (PROEX 365.3/21).

### Chick chorioallantoic membrane assays

Chick chorioallantoic membrane (CAM) assays were performed as previously described 41 using premium specific pathogen-free, 9-day-old embryonated chicken eggs (Gibert farmers). U87/U87shC3G (1 × 10^6^) cells diluted in PBS with Matrigel were inoculated. After 7 days, tumors were excised, measured and fixed with 4% PFA for further analysis.

### Analysis of paraffin-embedded tumor samples

Tumors from orthotopic xenograft and CAM assays were embedded in paraffin, cut into 8 µm sections and processed as previously described 29. After deparaffinization and rehydration, antigen retrieval with citrate buffer (10 mM, pH = 6) was performed. Samples were then permeabilized with 0.3% Triton X-100, blocked with 1% normal goat serum-3% BSA-PBS for 30 min at RT and incubated overnight at 4 °C with anti-PKM2 antibody (Cell signaling 3198S) diluted in blocking solution (1:50). After washing, sections were incubated with secondary antibody (goat anti-rabbit-Alexa 555; Invitrogen A32732; 1:200) and DAPI (Sigma D9542; 1 μg/mL) for 2 h at RT in dark. Finally, sections were mounted with mounting medium (Invitrogen P36930).

### PTBP1 and PKM2 silencing

Predesigned siRNAs against human *PTBP1* were obtained from Dharmacon (L-003528-00-0005). U87 shControl and shC3G were dissociated and seeded at 2.5 x 10^5^ cells per 60-mm culture dish. Cells were transfected with 50 nM of siRNA using lipofectamine RNAiMAX (Invitrogen 13778075) in Opti-MEM medium (Gibco 31985047), according to manufacturer's instructions. After 72 h of incubation, cells were harvested for western blot and mRNA expression analyses.

Custom Silencer Select siRNA targeting human *PKM2* were synthesized by Thermo Fisher Scientific. The siRNA sequences used were the following (5′→3′): CCAUAAUCGUCCUCACCAA, GCCAUAAUCGUCCUCACCA, and AGGCAGAGGCUGCCAUCUA. 12Φ12 shControl and shC3G cells were dissociated and seeded at a density of 2 × 10⁵ cells per 60-mm culture dish. Cells were transfected with 50 nM siRNA using Lipofectamine RNAiMAX (Invitrogen, 13778075) in Opti-MEM medium (Gibco, 31985047), according to the manufacturer's instructions. A second round of transfection was performed 24 h after the initial transfection. Cells were harvested 48 h after the second transfection for western blot, and functional assays.

### Limited dilution assays (LDA)

LDA is a dose-response experimental method to define the frequency of CSCs in a tumor tissue 42. For this experiment, U87 and 12Ф12 cells were plated in 96-well non-adherent plates at different densities (200, 100, 50, 25, 13, 6, 3 and 1 cell per well) using neurosphere medium. After one or two weeks, neurosphere generation was evaluated: wells where there was at least one neurosphere were counted as positive.

These results were visualized by dose-response graphs representing the number of cells seeded per well (axis X) *vs.* the logarithm of the fraction of negative wells (non-responding cells) (axis Y). Consequently, the line slope, represented in the graph by a continuous line, is directly proportional to the auto-renewal capacity of cells. A higher slope value indicates a higher fraction of cells with auto-renewal capacity. Discontinuous lines indicate the 95% confidence intervals and dots represent the values for each cell number.

### Viability assays

Cell viability in response to compound 3K (SelleckChem S8616) was assessed using crystal violet staining for adherent cells, and cell counting for neurospheres.

For adherent cells, 3 × 10^5^ U87 cells or 1.5 × 10^5^ 12Ф12 cells per well were seeded in a 24-well plate for 24 h followed by treatment with increasing concentrations of compound 3K (0-15 μM for U87 cells and 0-10 μM for 12Ф12 cells) for 24, 48, and 72 h. Cells were then stained with 0.05% crystal violet (Panreac 251762.1606) to evaluate viability.

For neurospheres, 5.5 × 10^5^ 12Ф12 cells were seeded in non-adherent 60-mm dishes with neurosphere medium supplemented with compound 3K (2.5 and 5 μM). Culture media was renewed every 3 days with fresh inhibitor-containing media. After 7 days, representative images of the spheres were acquired using Eclipse TE300 microscope (Nikon), and neurospheres were dissociated with Accutase™ (Corning 25058CI) for cell counting.

### Statistical analysis

Data were represented as the mean values ± S.E.M. (standard error of the mean) of at least, 3 independent experiments. Unpaired Student's t-test was used to compare two experimental groups under normal data distribution. One-way or two-way ANOVA analyses were used to compare more than two groups with one or two variables, respectively, followed by a multiple comparison Bonferroni test. All statistical analyses were carried out with GraphPad Prism Software version 8.0.1. Statistical significance was considered when p value ≤0.05.

## Results

### C3G down-regulation in GBM cells upregulates PKM2 and LDHA expression reprogramming metabolism

**Figure 1:**
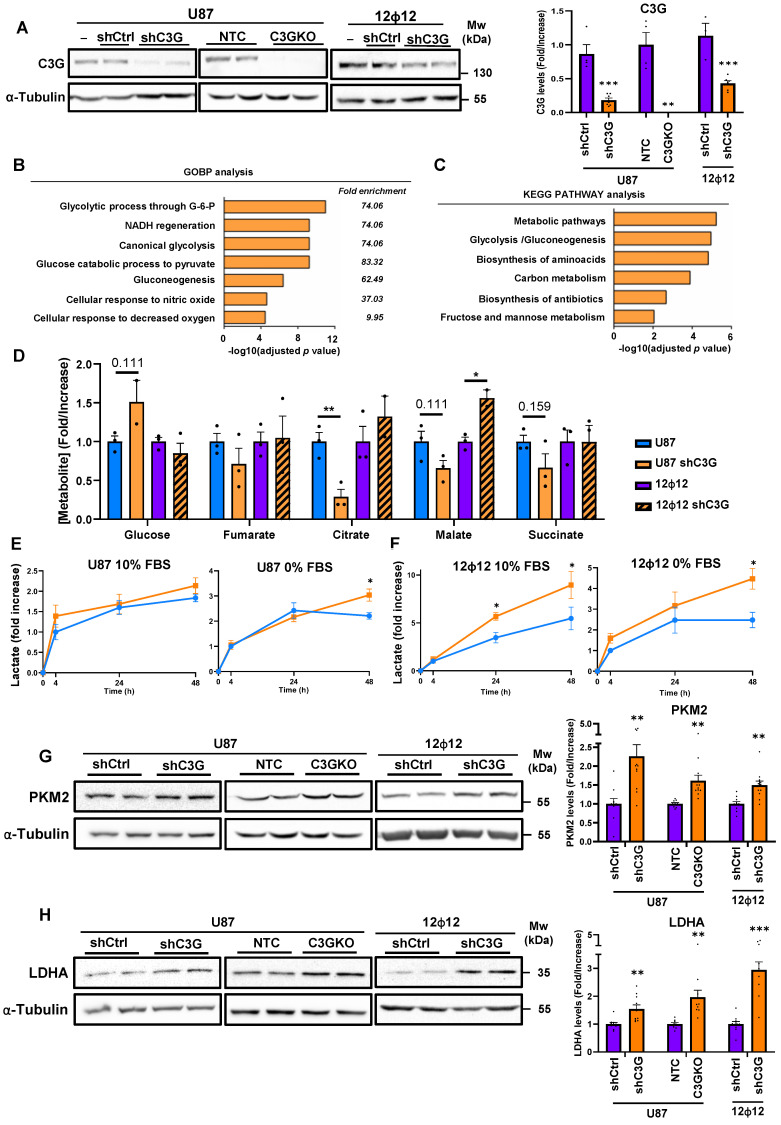
** C3G silencing or knockout in GBM cells increases the expression of glycolytic enzymes.** Non-silenced U87 (U87 or U87shControl (U87shCtrl)) and 12Ф12 (12Ф12 or 12Ф12shControl (12Ф12shCtrl)), C3G-silenced U87 (U87shC3G) and 12Ф12 (12Ф12shC3G), as well as U87 C3G knockout (U87C3GKO) cells and their control (U87NTC) were used. **A)** Western blot analysis of C3G protein levels normalized with α-Tubulin (left panel). Histograms show the quantification (main value ± S.E.M.) of the ratio C3G/α-Tubulin (right panel) of different western blots (n=3-6). **B-C)** GOBP (gene ontology biological processes) enrichment **(B)** and KEGG Pathway terms** (C)** determined by DAVID software using the list of IDs overrepresented proteins in U87shC3G compared to parental U87cells according to a proteome-wide analysis (n=4). **D)** Metabolomic analysis of glucose and TCA metabolites. Histograms show the fold increase in their concentration (n=3). **E-F)** Extracellular lactate levels in U87 **(E)** and 12Ф12 cells **(F)** maintained in a medium supplemented with 10% or 0% FBS. Graphics show the fold increase in lactate levels at 4, 24 and 48 h (n=3-5). **G)** Western blot analysis of PKM2 (upper panel) and LDHA (lower panel) protein levels normalized with α-Tubulin. Histograms (right panel) show the quantification (main value ± S.E.M.) of PKM2/α-Tubulin and LDHA/ α-Tubulin ratio of different western blots (n=7-11). *p≤0.05; **p≤0.01 and ***p≤0.001 compared to non-silenced cells.

Considering the relevant function of C3G in GBM controlling invasion and tumor aggressiveness previously identified in our group 37, the key role of metabolism reprogramming in these tumors 12,13, and the function of C3G 38, and its target, Rap1, regulating glucose metabolism in the liver 39, we evaluated the potential contribution of C3G to rewire GBM metabolism. To do it, two cell lines were used, U87, which is more differentiated, and 12Φ12 cells with a stem-like phenotype 40. In both cases, they were genetically modified to obtain non-silenced (shControl), C3G-silenced U87 (shC3G), U87KO and its control (U87NTC), as well as non-silenced and C3G-silenced 12Φ12 cells. In addition, key experiments were performed in HCO1 cells, also derived from a patient tumor. C3G protein levels were highly down-regulated in C3G-silenced- U87, 12Φ12 (Figure 1A) and HCO1 cells (Figure S1A) and were undetectable in U87C3GKO cells (Figure 1A).

First, we performed a proteome-wide analysis to compare the protein profile of non-silenced U87 versus C3G-silenced U87 cells to identify potential changes in proteins from different metabolic pathways. We found several proteins that were either upregulated in non-silenced U87 cells (Table S1) or in C3G-silenced U87 cells (Table S2), which were ranked from higher to lower score (*column 3*) considering the number of matched peptides (*column 4*) and the fold change between non-silenced and C3G-silenced cells (*column 5*) in each case. Only proteins with statistically significant changes were considered (*column 6*). Among all these proteins there were various enzymes from the glycolytic pathway, including PKM. Using these data and Panther software, several enrichment analyses were performed to determine which GO (*gene ontology*) terms were regulated by C3G levels. Diverse metabolism-associated GO terms were detected by GOBP (*GO biological processes*) analysis (Figure 1B) such as “Glycolytic process through glucose-6-phosphate”, “Canonical glycolysis” and “Glucose catabolic process to pyruvate”, which were overrepresented in C3G-silenced U87 cells.

KEGG PATHWAY analysis using DAVID software also identified an enrichment in metabolic pathways such as “Canonical glycolysis”, “Glycolytic process” or “Glycolysis/Gluconeogenesis”) (Figure 1C) in U87shC3G cells. Therefore, all these data support that C3G downregulation could favor glycolysis.

**Figure 2:**
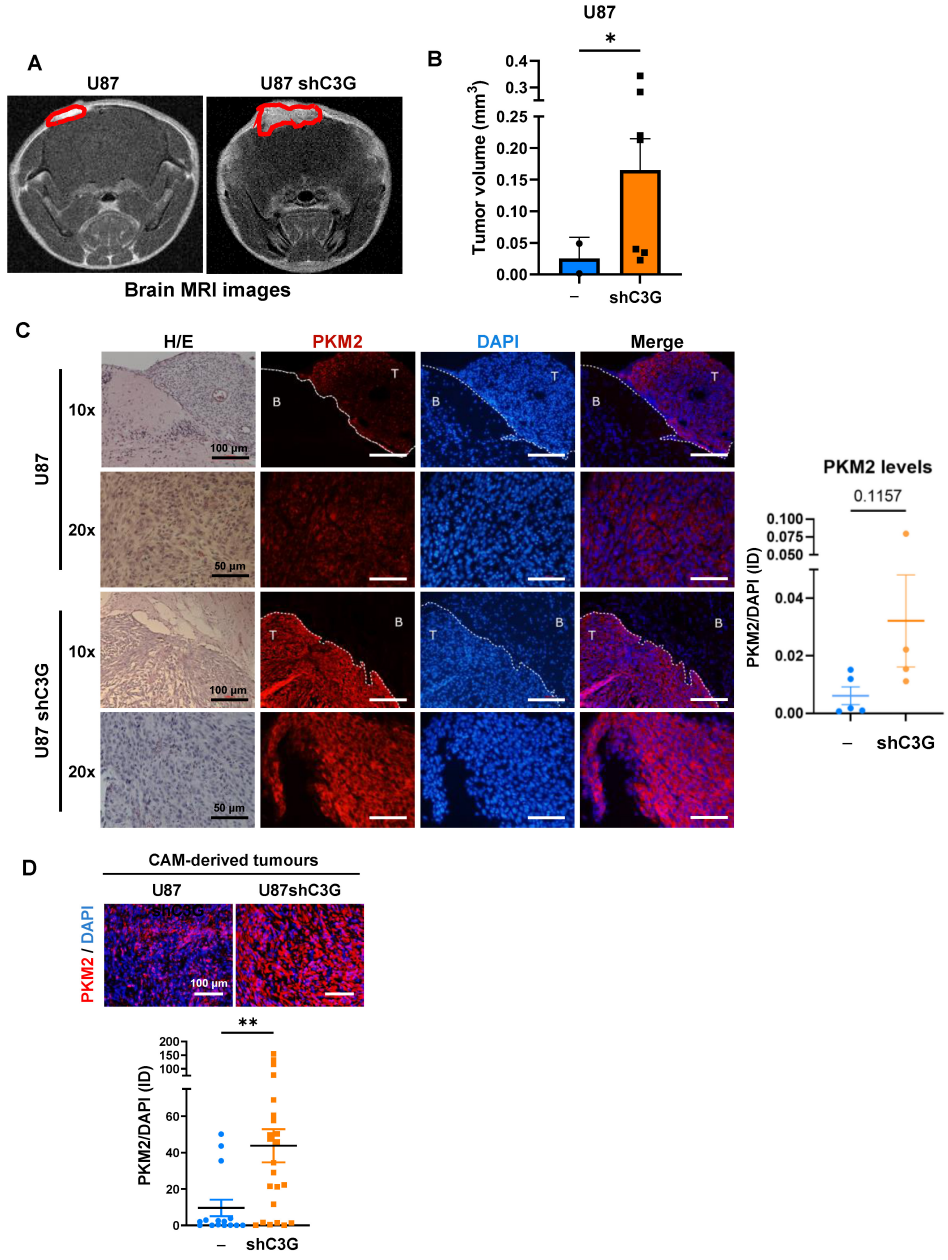
** PKM2 is upregulated in tumors generated by GBM cells with C3G downregulation. A)** Representative magnetic resonance imaging (MRI) images showing orthotopic tumors generated by C3G-silenced cells (U87shC3G) and parental U87 cells (U87), n=3. **B)** Histogram showing the volume of these tumors in mm^3^. PKM2 immunofluorescence analysis of PKM2 in brain orthotopic tumors (n=4-5)** (C)** and in tumors generated in CAM assays generated by C3G-silenced cells (U87shC3G) and parental U87 cells (U87) 7 days after injection of the cells (n=15-23)** (D)**. Graphics showing fluorescence intensity of PKM2 versus DAPI. Scale bars (100 μm for 10X images and 50 μm for 20X images). *p≤0.05 and ***p≤0.001 compared to non-silenced cells.

To further understand the potential function of C3G regulating GBM cell metabolism, the effect of C3G silencing on the cellular concentration of glucose and different TCA metabolites in U87 and 12Φ12 cells was analyzed. It was detected that there was a tendency to increase intracellular glucose levels, a significant reduction in citrate concentration and a trend to decrease malate and succinate in U87shC3G compared with non-silenced U87 cells (Figure 1D). This might be due to the incorporation of citrate to fatty acid synthesis through its previous conversion to acetylCoA. This is supported by the higher levels of ACC (acetylCoA carboxylase) and FAS (Fatty acid synthase), but lower of P-ACC (inactive form) found in U87shC3G cells, and no changes in β-oxidation enzyme CPT1A (Carnitine palmitoyl transferase 1A) (Figure S2). However, in 12Φ12 cells, malate concentration was higher in C3G silenced cells (Figure 1D) and only FAS levels increased in C3G-silenced cells, while a trend to increase CPT1A was observed (Figure S2). Therefore, TCA behavior was apparently different in U87 and 12Φ12 cells upon C3G silencing, which could be related with differences in fatty acid metabolism. Then, the presence of lactate in the media was also quantified, detecting an increase upon C3G silencing in both cell lines (Figure 1E-1F) under the different culture conditions (with 10% FBS or without serum). This suggests an enhancement of glycolysis upon C3G silencing in GBM cells. This is usually mediated by the upregulation of PKM2 isoform and LDHA, which favor pyruvate and lactate generation 43,44. In fact, a significant increase in PKM2 and LDHA protein levels was observed in C3G-silenced U87, 12Φ12 and HCO1 cells, and in U87C3GKO cells (Figure 1G-1H and Figure S1B) when compared with their respective control cells (non-silenced or non-KO (NTC) for C3G). The higher levels of PKM2 in U87shC3G, 12Φ12shC3G and U87C3GKO were also corroborated by immunofluorescence analysis (Figure S3A). Moreover, re-expression of either C3G full length (C3GFL) or C3G lacking the catalytic domain (C3GΔCat) in U87C3GKO cells partially prevented this PKM2 upregulation (Figure S3B). In addition, in orthotopic GBM tumors generated by C3G-silenced U87 cells, which had a higher volume (Figure 2A-2B), PKM2 was upregulated as well (Figure 2C). PKM2 protein levels were also increased in (CAM)-derived tumors from U87shC3G cells compared with tumors from non-silenced U87 cells (Figure 2D). Hence, C3G silencing or deletion promotes PKM2 induction*, in vitro* and *in vivo.*

### C3G down-regulation in GBM cells enhances cellular respiratory capacity and glycolysis

**Figure 3:**
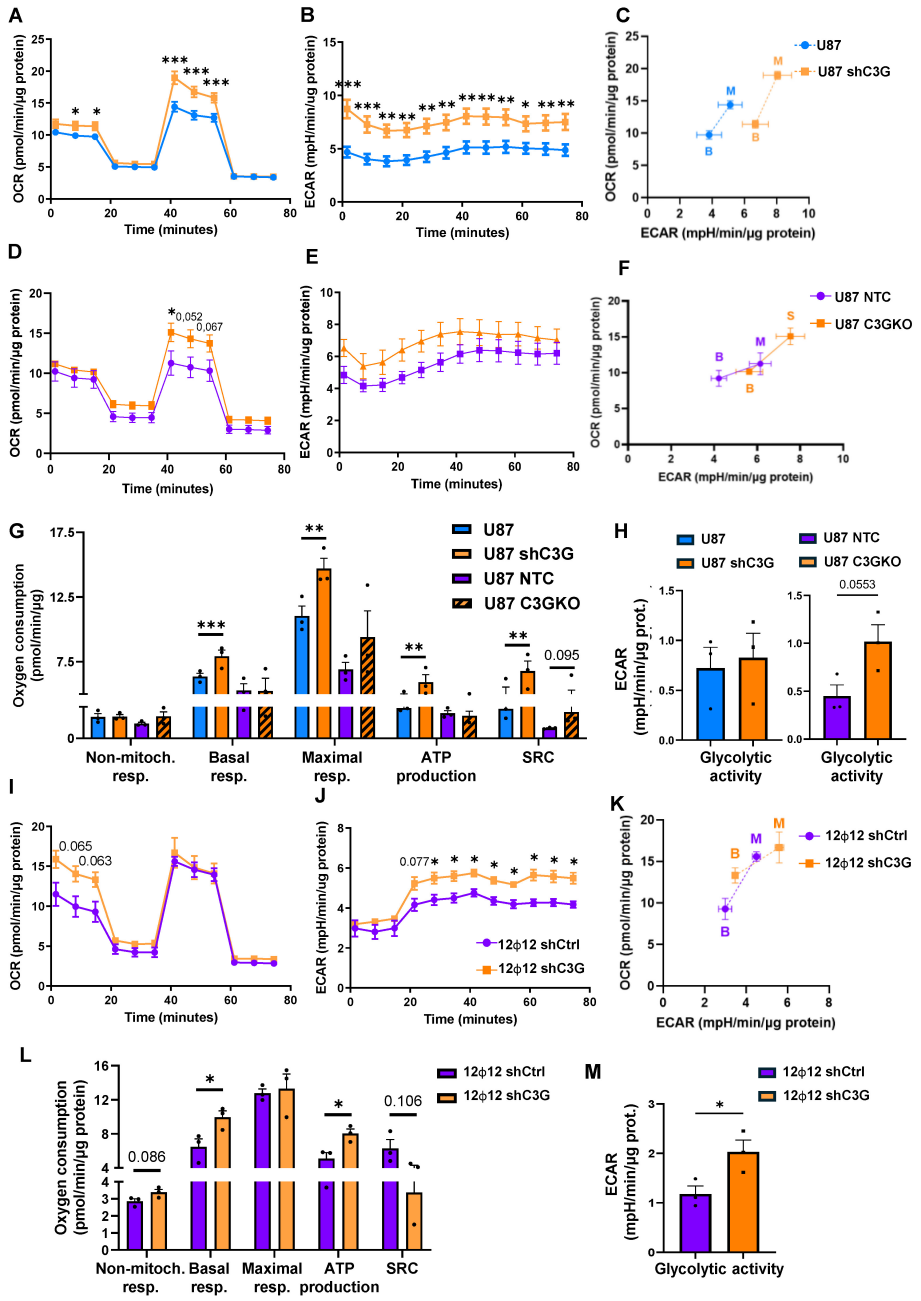
** Mitochondrial respiration and glycolysis are enhanced by C3G down-regulation or depletion in GBM cells.** Non-silenced U87 and 12Ф12 (12Ф12shControl (12Ф12shCtrl)), C3G-silenced U87 (U87shC3G) and 12Ф12 (12Ф12shC3G) cells, U87 C3G knockout (U87C3GKO) cells and their control (U87NTC) were used. **A-K)** Analysis of metabolic activity using Seahorse technology. **A-C)** Graphics showing **(A)** Oxygen consumption rate (OCR), **(B)** extracellular acidification rate (ECAR) time course and** (C)** cell energy phenotype data (relationship between OCR and ECAR, with dots representing maximal (M) and basal (B) respiration) of U87 and U87shC3G cells (n=3). **D-F)** Graphics showing **(D)** OCR, **(E)** ECAR time course and **(F)** cell energy phenotype data of U87NTC and U87C3GKO cells are shown (n=3). **G)** Histogram showing the quantification of non-mitochondrial respiration (non-mitoch. resp) and mitochondrial respiratory parameters: basal respiration (basal resp.), maximal respiration (maximal resp.), ATP synthesis and spare respiratory capacity (SRC) in U87 cells. **H)** Histograms showing the quantification (mean value ±S.E.M.) of the glycolytic activity of U87 vs U87shC3G cells and U87NTC vs U87C3GKO cells (n=3). **I-J)** Graphics showing **(I)** OCR, **(J)** ECAR time course and **(K)** cell energy phenotype data of 12Ф12shCtrl and 12Ф12shC3G cells are shown (n=3). **L)** Histogram showing the quantification of non-mitochondrial respiration and mitochondrial respiratory parameters in 12Ф12 cells (n=3). **M)** Histograms showing the quantification (mean value ±S.E.M.) of the glycolytic activity 12Ф12shControl vs 12Ф12shC3G cells (n=3). *p≤0.05; **p≤0,01 and ***p≤0.001 compared to non-silenced or NTC cells.

To further understand how cellular metabolism was altered by C3G downregulation, real-time measurements of GBM cell metabolism, focusing on mitochondrial respiration and glycolysis, were performed using Seahorse equipment. As shown in Figure 3A-3H, basal and maximal oxygen consumption rate (OCR), as well as extracellular acidification rate (ECAR) increased in U87shC3G and U87C3GKO cells compared with their respective controls (non-silenced or NTC U87 cells) leading to a more glycolytic cell energy phenotype (Figure 3C, 3F and 3H). This indicates that mitochondrial OXPHOS and glycolysis are enhanced by C3G downregulation or knockout. Moreover, basal and maximal mitochondrial respiration, mitochondrial ATP production and the spare respiratory capacity were also increased in U87shC3G cells (Figure 3G). However, the behavior of 12Φ12 cells was slightly different compared to U87 cells. C3G silencing enhanced basal respiration rate, ECAR and mitochondrial ATP synthesis, but not the spare respiratory capacity in 12Φ12shC3G, leading to a more glycolytic cell energy phenotype than non-silenced cells (Figure 3I-3M). In addition, non-mitochondrial respiration tended to be increased in 12Φ12shC3G cells compared to non-silenced 12Φ12 cells (Figure 3L). Therefore, basal mitochondrial OXPHOS is increased by C3G silencing in both U87 and 12Φ12, and in C3G knockout U87 cells, while maximal mitochondrial is only enhanced in C3G-silenced and knockout U87 cells. This difference in the maximal mitochondrial respiration between U87shC3G/C3GKO and 12Φ12shC3G cells could be due to the existence of more mitochondria in U87 cells, based on the higher intensity of MitoTracker staining (Figure S4A-S4B). Besides, C3G deletion in U87 and C3G silencing in 12Φ12 cells increased mitochondrial content. On the other hand, glycolysis was favored in U87shC3G, U87C3GKO and 12Φ12shC3G, in agreement with the enhanced lactate secretion, although according to Seahorse data it was only significantly increased in 12Φ12shC3G cells as compared to controls.

### C3G downregulation promotes PTBP1-mediated *PKM* alternative splicing enhancing PKM2 expression in GBM cells

**Figure 4:**
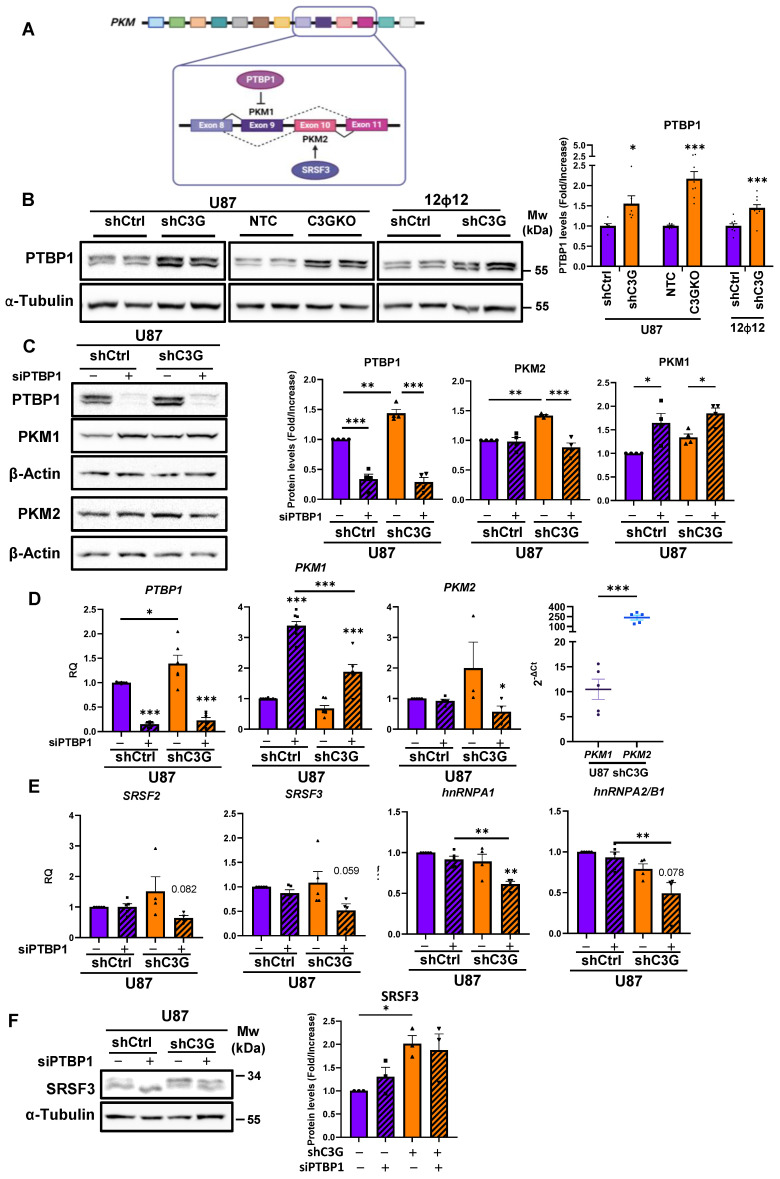
** C3G downregulation induces PKM2 expression through regulation of *PKM* splicing by the splicing factor PTBP1 in GBM cells.** Non-silenced U87 (U87shControl (U87shCtrl)) and 12Ф12 (12Ф12shControl (12Ф12shCtrl)), C3G-silenced U87 (U87shC3G) and 12Ф12 (12Ф12shC3G), as well as U87 C3G knockout (U87C3GKO) cells and their control (U87NTC) were used. **A)** Scheme showing the regulation of *PKM* splicing by PTBP1 and SRSF3 splicing factors. PTBP1 inhibits PKM1 expression through the exclusion of exon 9, and SRSF3 favors the inclusion of exon 10, promoting PKM2 expression. **B)** Western blot analysis of PTBP1 protein levels normalized with α-Tubulin. The histogram shows the quantification (mean value ±S.E.M.) of the ratio PTBP1/α-Tubulin of different western-blots (n=6). **C)** Western blot analysis of PTBP1, PKM1 and PKM2 protein levels normalized with α-Tubulin upon transient silencing of PTBP1 in U87shCtrl and U87shC3G cells. Histograms showing the quantification (mean value ±S.E.M.) of the ratio PKM2/α-Tubulin and PKM1/α-Tubulin of different western-blots (n=4). **D)** Quantification of *PTBP1*, *PKM1* and *PKM2* mRNA levels by RT-qPCR upon transient silencing of PTBP1 in U87shCtrl and U87shC3G cells and the levels of PKM1 vs PKM2 mRNAs in U87shC3Gcells (last right graphic) (n=3-5). **E)** Quantification of *SRSF2, SRSF3, hnRNPA1* and *hnRNPA1/B1* mRNA levels by RT-qPCR upon transient silencing of PTBP1 in U87shCtrl and U87shC3G cells (n=4-5). **F)** Western blot analysis of SRSF3 protein levels normalized with α-Tubulin upon transient silencing of PTBP1 in U87shCtrl and U87shC3G cells. Histogram shows the quantification (mean value ±S.E.M.) of the ratio SRSF3/α-Tubulin of different western-blots (n=3). *p≤0.05; **p≤0.01 and ***p≤0.001 compared to non-silenced cells.

The metabolic shift towards glycolysis identified in C3G-silenced and -knockout GBM cells was associated with an upregulation of PKM2 protein levels. Therefore, C3G is modulating PKM2 expression, which is mainly regulated by *PKM* RNA splicing 44. *PKM* RNA can lead to PKM1 and PKM2 isoforms through alternative splicing, which is regulated by various splicing factors including PTBP1, SRSF3, hnRNPA1 and hnRNPA2/B1 (Figure 4A) 46.

Moreover, C3G is known to regulate RNA splicing through modulation of several splicing factors such as SRSF2 47. Hence, we analyzed whether PTBP1 expression was controlled by C3G in GBM cells. We found that PTBP1 protein levels increased in C3G-silenced U87, 12Φ12 and HCO1 cells and in U87C3GKO cells vs their controls (Figure 4B and Figure S5A), which was partially reverted by re-expression of C3G full length (C3GFL) or C3G lacking catalytic domain (C3GΔCat) in U87C3GKO cells (Figure S5B). Furthermore, transient PTBP1 gene silencing using siRNAs efficiently reduced *PTBP1* protein levels and raised PKM1 protein levels in both U87shControl and U87shC3G cells, impairing PKM2 upregulation in U87shC3G cells (Figure 4C). These changes in PKM1 and PKM2 protein levels correlated with the expression of *PKM1* and *PKM2* mRNAs upon *PTBP1* silencing (Figure 4D). Notably, PTBP1 silencing abolished the upregulation of *PKM2* mRNA in U87shC3G cells and enhanced *PKM1* mRNA expression in U87shControl cells. In addition, PTBP1 silencing in C3G knockdown U87 cells led to a reduction in *SRSF2*, *SRSF3*, *hnRNPA1* and *hnRNPA2/B1* mRNA levels, although no effect of C3G silencing was observed (Figure 4E). However, SRSF3 protein levels were higher in C3G-silenced U87 cells and tended to decrease upon PTBP1 silencing (Figure 4F). These results indicate that the increase in PTBP1 expression induced by C3G-silencing would promote PKM2 upregulation through regulation of *PKM* splicing. SRSF2, SRSF3, hnRNPA1 and hnRNPA2/B1 could also contribute to the effect of PTBP1 on PKM2 expression, particularly, SRSF3.

Concerning the mechanism mediating C3G effect on PTBP1, we found that ERKs inhibition prevented the increase in PTBP1 protein levels induced by C3G silencing in 12Φ12 cells (Figure S5C). Therefore, ERKs mediate PTBP1 expression induced by C3G-downregulation.

### C3G silencing enhances stemness and tumor initiating capacity in GBM cells through a mechanism dependent on PKM2

**Figure 5:**
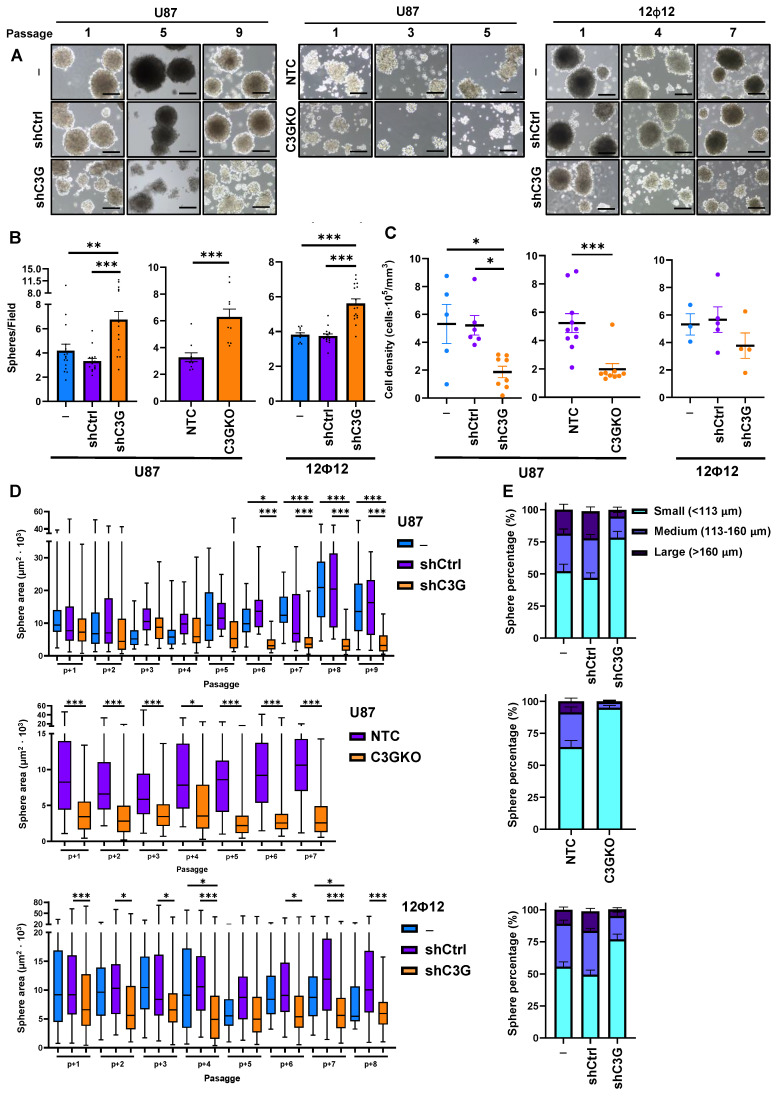
** C3G silencing or its depletion in GBM cells promotes the generation of neurosphere-like structures.** Non-silenced U87 (U87shControl (U87shCtrl)) and 12Ф12 (12Ф12shControl (12Ф12shCtrl)), C3G-silenced U87 (U87shC3G) and 12Ф12 (12Ф12shC3G), as well as U87 C3G knockout (U87C3GKO) cells and their control (U87NTC) were used. **A)** Representative phase-contrast microscope images of spheres at different passages as indicated. Scale bar = 100 μm. **B)** Histograms showing sphere number per microscope field (n=10-16). **C)** Graphics showing cell density within the spheres expressed as number of cells per mm^3^ (n=3-10). **D)** Histograms showing sphere area expressed as μm^2^x10^3^. **E)** Histograms showing the percentage of spheres with low, medium and large size as indicated. *p≤0.05; **p≤0.01 and ***p≤0.001 compared to non-silenced or NTC cells.

As mentioned before, glycolysis plays a pivotal role in GBM 14, as well as its product, lactate, which has multifaceted functions aimed to support GBM tumor growth and progression 48,49. Enhanced glycolysis is associated with high levels of PKM2 14, which also promotes stemness and self-renewal in GBM 19. Therefore, based on the enhanced glycolysis and PKM2 expression observed in C3G-silenced U87 and 12Φ12 GBM cells, we hypothesized that C3G downregulation could increase stemness and tumor initiating capacities in GBM cells. Hence, we analyzed the sphere-forming ability in U87, 12Φ12 and HCO1 GBM cells upon C3G silencing or depletion. As shown in Figures 5A, 5B and S6, the number of spheres per microscope field was significantly higher in U87shC3G, U87C3GKO, 12Φ12shC3G and HCO1shC3G cells at the different passages compared with their respective controls. Moreover, expression of either C3GFL or C3GΔCat in U87C3GKO cells prevented this increase in sphere formation (Figure S7A). However, the spheres displayed lower cell density (Figure 5C), and reduced size (Figure 5D, 5E and S6C) when C3G was downregulated or absent. Thus, sphere area was significantly reduced in C3G-silenced U87, 12Φ12 and HCO1 cells, and in C3G knockout U87 cells. This size reduction was further supported by CellTrace experiments, which revealed dye accumulation in C3G-silenced 12Φ12 spheres after 7 days due to their decreased proliferation (Figure S7B). Consistently, the percentage of spheres with a small size increased upon C3G silencing in both U87 and 12Φ12 cells and in C3G-knockout U87 cells, while decreasing those of large sizes at all passages (Figure 5E).

**Figure 6:**
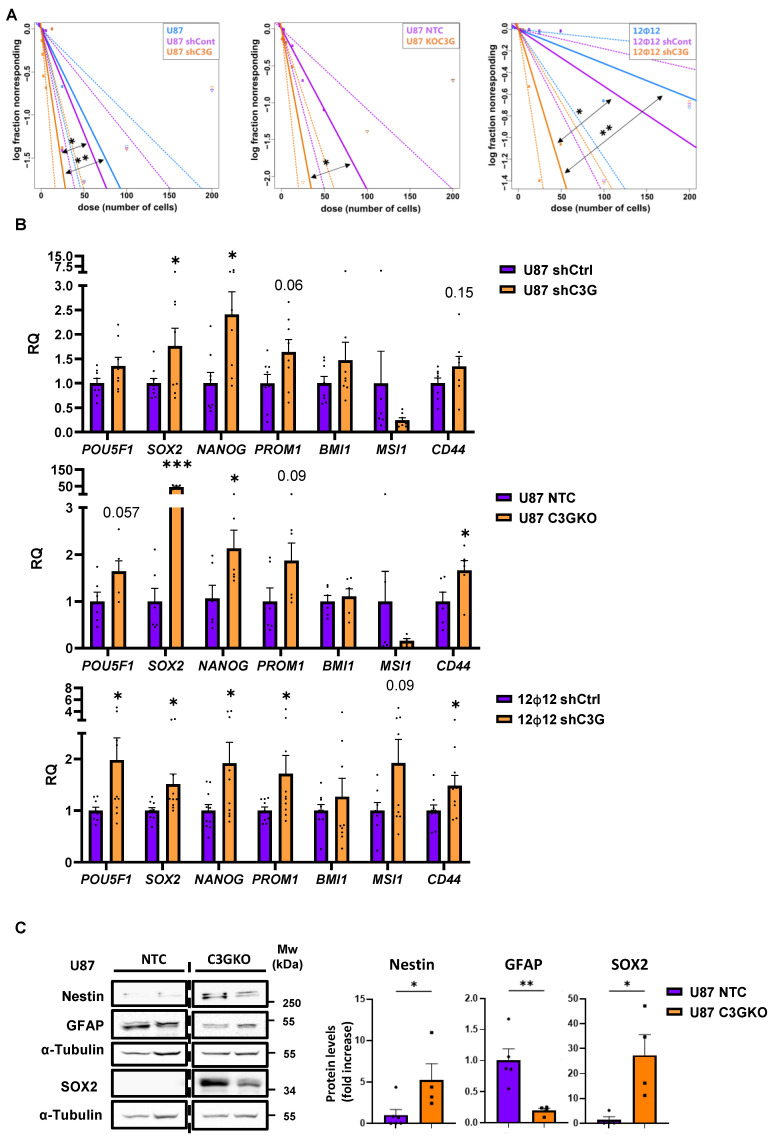
** GBM stemness and tumor initiating capacity is increased upon C3G silencing or depletion.** Non-silenced U87 (U87shControl (U87shCtrl)) and 12Ф12 (12Ф12shControl (12Ф12shCtrl)), C3G-silenced U87 (U87shC3G) and 12Ф12 (12Ф12shC3G), as well as U87 C3G knockout (U87C3GKO) cells and their control (U87NTC) were used. **A)** Limited dilution assays in sphere passage 2. Results are visualized by dose-response graphs representing the number of cells seeded per well (axis X) *vs.* the logarithm of the fraction of negative wells (non-responding cells) (axis Y). The line slope, represented by a continuous line, is directly proportional to the auto-renewal capacity of cells. Discontinuous lines indicate the 95% confidence intervals and dots represent the values for each cell number. 12Ф12 data were obtained after 1 week and U87 data after 2 weeks. **B)** Histograms showing the quantification of mRNA levels of stemness markers by RT-qPCR on passage 1 (n=6-9). **C)** Western-blot analysis of GFAP, Nestin, and SOX2 protein levels normalized with α-Tubulin in U87C3GKO and U87NTC cells. The histogram shows the quantification expressed as fold increase (n=4-5). *p≤0.05; **p≤0.01 and ***p≤0.001 compared to non-silenced or NTC cells (n=4-9).

GSCs are responsible for GBM recurrence due to its tumor initiating capacity 9,50. Hence, this ability was evaluated by doing a limiting dilution assay (LDA), a well-recognized experimental approach to quantify self-renewal and tumorigenic capacities 42. Dose-response graphs showing the number of cells seeded per well (axis X) *vs.* the logarithm of the fraction of non-responding cells (axis Y) are shown in Figure 6A for spheres in passage 2. According to the continuous line slope, which is proportional to cell self-renewal capacity, spheres formed by C3G-silenced U87 and 12Φ12 cells, and knockout U87 cells showed a significant increase in self-renewal capacity compared with their respective controls (Figure 6A). However, it should be noticed that the time required to form spheres in the case of U87 cells was much longer (2 weeks) than that of 12Φ12 cells (1 week).

The mRNA expression of stemness markers such as *POU5F1*, *SOX2*, *NANOG*, or *PROM1* also increased (Figure 6B) in C3G-silenced U87 and 12Φ12 cells, and knockout U87 cells. Moreover, the protein levels of GFAP, a marker of differentiated astrocytes, decreased in C3G knockout U87 cells, while protein levels of the stemness markers, Nestin and SOX2, increased (Figure 6C). These results support that C3G downregulation or deletion promotes GBM stemness and tumor initiating capacity. As mentioned before, glycolysis and PKM2 play a pivotal role in GBM cell proliferation and self-renewal 13,14. Therefore, we evaluated the relevance of PKM2 upregulation on cell viability and stemness capacity of C3G-silenced or knockout GBM cells using the PKM2 inhibitor 3K.

**Figure 7:**
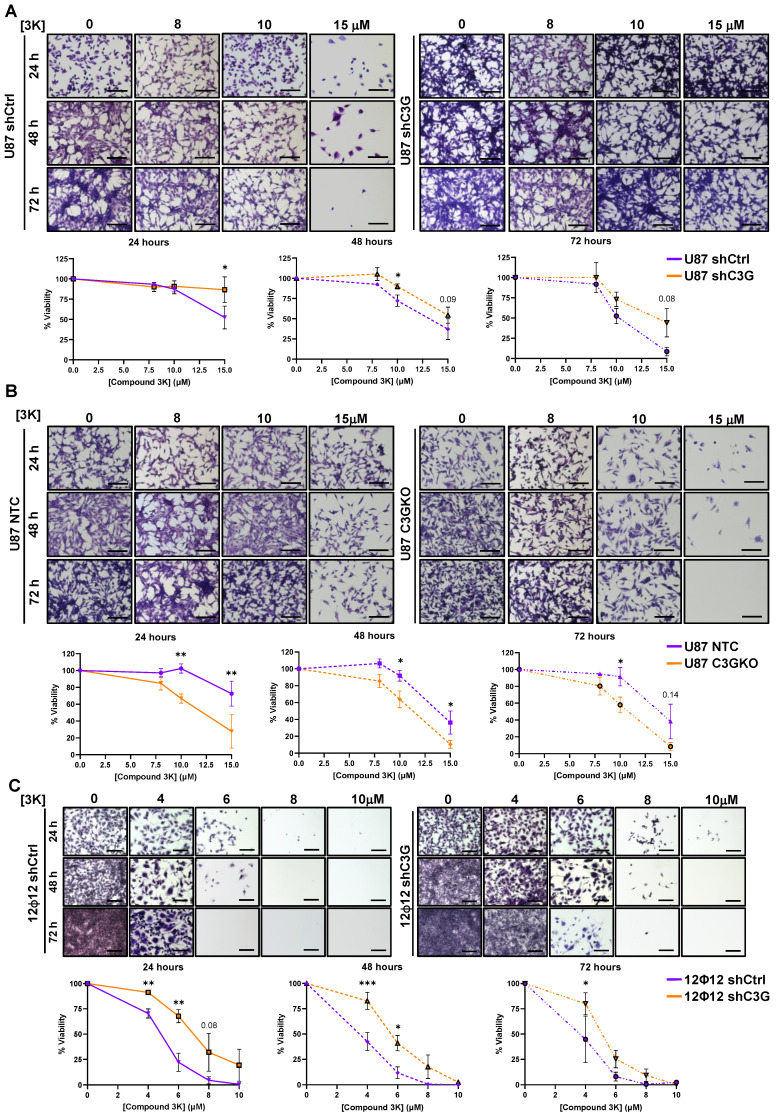
** PKM2 inhibition increases cell viability in C3G-silenced GBM cells while reducing viability of C3G-depleted cells.** Non-silenced U87 (U87shControl (U87shCtrl)) and 12Ф12 (12Ф12shControl (12Ф12shCtrl)), C3G-silenced U87 (U87shC3G) and 12Ф12 (12Ф12shC3G), as well as U87 C3G knockout (U87C3GKO) cells and their control (U87NTC) were used. Cells were treated with the PKM2 inhibitor, compound 3K, at the indicated concentrations or with DMSO (vehicle) for 24, 48 or 72 h. Cell viability was assessed by crystal violet staining. **A)** Effect of compound 3K on U87shCtrl and U87shC3G cells. **B)** Effect of 3K compound on U87NTC and U87C3GKO cells. **C)** Effect of compound 3K on 12Ф12shCtrl and 12Ф12shC3G cells. Upper panels, representative phase-contrast microscope images of stained cells; lower panels, graphics showing the percentage of cells at different times. Scale bar = 100 μm. *p≤0.05; **p≤0.01 and ***p≤0.001 compared to non-silenced or NTC cells (n=4).

**Figure 8:**
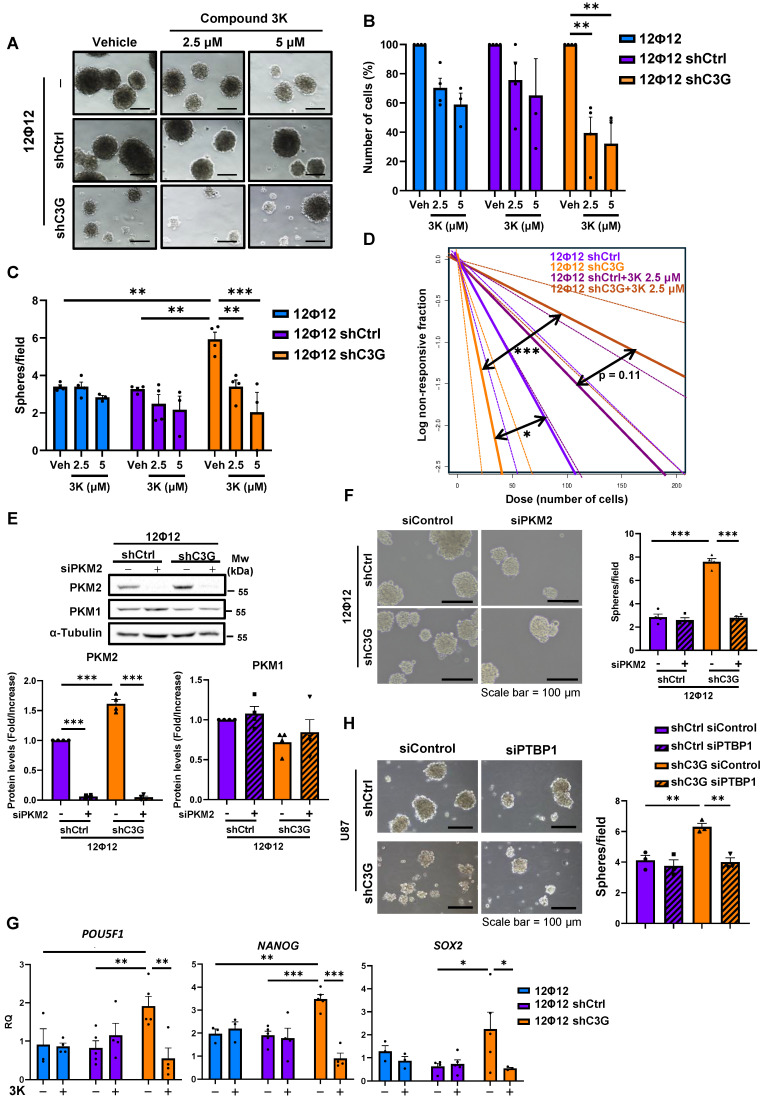
** PKM2 inhibition prevents the enhancement of stemness capacity induced by C3G down-regulation.** Non-silenced12Ф12 (12Ф12shControl (12Ф12shCtrl)) and C3G-silenced 12Ф12 (12Ф12shC3G) cells were used. Cells were treated with the PKM2 inhibitor, compound 3K, at the indicated concentrations or with DMSO (vehicle) for 72 h. **A)** Representative phase-contrast microscope images of spheres at passage 2. **B)** Cell viability in spheres. The histogram shows the number of cells within the spheres expressed as percentage (n=3-4). **C)** Histogram showing sphere number per microscope field (n=3-4). **D)** LDA assay. Results are visualized by dose-response graphs representing the number of cells seeded per well (axis X) *vs.* the logarithm of the fraction of negative wells (non-responding cells) (axis Y). The line slope, represented by a continuous line, is directly proportional to the auto-renewal capacity of cells. Discontinuous lines indicate the 95% confidence intervals and dots represent the values for each cell number. **E)** Western-blot analysis of PKM2 and PKM1 protein levels normalized with α-Tubulin upon transient silencing of PKM2 using siRNAs. Histograms showing the quantification (mean value ±S.E.M.) of the ratio PKM2/α-Tubulin and PKM1/α-Tubulin of different western-blots (n=4). **F)** Effect of PKM2 silencing on sphere-forming capacity in GBM cells. Sphere formation was evaluated in C3G-silenced (12Ф12shC3G) cells and control cells (12Ф12shControl) 12Ф12 cells. Representative phase-contrast microscope images of spheres at passage 1 (left panel). Histogram showing sphere number per field (right panel) (n=4). **G)** Effect of PKM2 inhibition by compound 3K at 2.5 μM stemness. Histograms showing the quantification of mRNA levels of stemness markers by RT-qPCR on passage 1 (n=4-5). **H)** Effect of PTBP1 silencing on sphere-forming capacity in GBM cells. Sphere formation was evaluated in C3G-silenced (U87shC3G) and control cells (U87shControl (U87shCtrl) U87 cells. Representative phase-contrast microscope images of spheres at passage 1 (let panel). Histogram showing sphere number per field (right panel) (n=3). Scale bar = 100 μm. *p≤0.05; **p≤0.01 and ***p≤0.001 compared to non-silenced cells.

Cell viability decreased upon treatment with 3K compound at concentrations of 10-15 µM in U87shControl, U87shC3G, U87NTC and U87C3GKO cells after 48-72 h of treatment (Figure 7A-7B). However, C3G-silenced U87 cells showed a trend to have higher survival rates than control cells, while U87C3GKO cells were more sensitive to PKM2 inhibitor, presenting a significant lower survival, even after 24 h. In agreement with this, apoptosis rates determined by flow cytometry (sub-G0/G1 cells) were higher in non-silenced U87 cells treated with 3K (10 µM) for 48 h (Figure S8A), and in U87C3GKO cells at 24h (Figure S8B). Notably, 12Φ12 cells were more sensitive to the PKM2 inhibitor, therefore, cell viability was progressively reduced at doses going from 2 μM up to 10 μM (Figure 7C), detecting a very low cell viability after 72 h of treatment. Moreover, significantly higher survival rates were observed in C3G-silenced 12Φ12 cells at 24, 48 and 72 h. Similarly, apoptosis (measured as active caspase 3 intensity) increased in non-silenced 12Φ12 cells treated with 3K (4 μM) for 48 h but not in C3G-silenced cells (Figure S8C). Therefore, GBM cells with low levels of C3G are less sensitive to PKM2 inhibition than control cells in terms of cell death, while cells lacking C3G showed the opposite effect in 2D cultures. This different behavior of C3G knockout and silenced GBM cells might be due to the differences in C3G protein levels. Low levels of C3G (20-30%) would be enough to enhance survival of GBM cells treated with 3K in 2D cultures, while total absence of C3G would not allow survival. Curiously, non-tumor cells (lung fibroblasts) showed a trend of a transiently decrease in cell viability at 24 and/or 48h with doses of 2.5 μM or 5 μM of 3K compound (Figure S9A), while survival increased at 72 h even with a 5 μM dose, which was able to kill around 70% of non-silenced 12Φ12 cells. This points to low toxicity of 3K in healthy cells. Hence, it was next analyzed the effect of PKM2 inhibitor on stemness capacity in C3G-silenced and non-silenced 12Φ12 cells. First, cell viability within the spheres was evaluated finding a significant reduction in cell survival only within spheres generated by C3G-silenced 12Φ12 cells treated with PKM2 inhibitor at doses of 2.5-5 μM (Figure 8A-8B). Moreover, the capacity to form spheres was also significantly reduced by treatment with 3K inhibitor in C3G-silenced, but not in non-silenced 12Φ12 cells (Figure 8C). In agreement with this, tumor initiating capacity, determined by LDA, decreased in C3G-silenced 12Φ12 cells treated with PKM2 inhibitor at doses of 2.5-5 μM (Figure 8D). All these results indicate that PKM2 upregulation induced by C3G silencing favors a stemness phenotype in GBM cells. This was also supported by data derived from genetically downregulation of PKM2 using siRNA in 12Φ12 cells, showing that transient PKM2 silencing impaired the increase in sphere forming capacity induced by C3G-silencing in 12Φ12 cells (Figure 8E-8F). Moreover, 3K inhibitor prevented the increase in the expression of the stemness markers *POU5F1*, *NANOG* and *SOX2* induced by C3G silencing (Figure 8G). Therefore, PKM2 would be a key mediator of C3G to regulate GBM cell stemness and tumor initiating capacity. Furthermore, considering that PTBP1 mediates the effect of C3G downregulation on PKM2, it was determined the impact of transient PTBP1 silencing on GBM cell stemness. As shown in Figure 8H, sphere forming capacity was reduced upon PTBP1 silencing, supporting that PTBP1/PKM2 axis plays a pivotal role mediating C3G effect on GBM cell stemness.

### Metabolic functions of PKM2 contribute to enhance stemness upon C3G downregulation

Since PKM2 is not only involved in metabolism but also in the regulation of gene expression in the nucleus 60, it was explored whether PKM2 actions on GBM cell stemness were dependent on metabolic changes. First, the effect of PKM2 inhibition by 3K on lactate production was determined. The results showed a decrease in lactate levels in the culture media upon treatment with 3K (Figure S9B) in both C3G-silenced and non-silenced 12Φ12 cells, which tend to be more pronounced in 12Φ12shC3G cells. This suggests a contribution of metabolism to PKM2-induced stemness. To further characterize it, sphere forming assays were performed in the absence or presence of pyruvate in the culture medium. As shown in Figure S9C, when pyruvate was absent there was only a tendency to increase the number of spheres in C3G-silenced 12Φ12 cells, that was blocked by 3K compound. In contrast, the addition of pyruvate to the medium (standard condition) accentuated sphere forming capacity in C3G-silenced 12Φ12 cells, which was blocked by 3K. Therefore, the enhanced stemness induced by C3G downregulation mediated by PKM2 seems to be mainly dependent on PKM2 metabolic functions, although a contribution of PKM2 as a direct regulator of gene expression in the nucleus cannot be discarded as PKM2 was present in both nucleus and cytosol in C3G-silenced 12Φ12 cells according to confocal analysis (Figure S10A). Total PKM2 levels significantly increased in C3G-silenced 12Φ12 cells, being the nuclear fraction around 45% of total PKM2. However, in U87 cells, PKM2 was mainly present in the cytosol, even in C3G-silenced cells, in which total levels of PKM2 also increased (Figure S10B).

## Discussion

We have previously demonstrated that C3G expression is downregulated in GBM cells and tumors. Moreover, low levels of C3G or its absence enhance migration and invasion through the induction of an epithelial-mesenchymal transition-like process and the hyper-activation of RTKs like FGFR1. C3G knockdown was also shown to increase tumor volume in *in vivo* CAM assays, while decreasing GBM cell proliferation 37.

In the present study, we now uncover a new role for C3G in GBM metabolism. Specifically, C3G downregulation or depletion enhances glycolysis and lactate generation, associated with PKM2 upregulation. This was consistently demonstrated using different approaches, including Seahorse-based metabolic profiling. Importantly, PKM2 mediates the potentiation of stemness and tumor initiating capacity of GBM cells induced by C3G knockdown through mechanisms, at least, partially depending on metabolic regulation. Hence, PKM2 inhibition with 3K highly decreased lactate production and blocked sphere forming capacity in C3G-silenced cells mainly when pyruvate was present in the medium. However, nuclear actions of PKM2 could contribute to promote stemness, particularly in 12Φ12 cells, where a significant fraction of PKM2 was localized in the nucleus.

These findings are particularly relevant given the essential role of PKM2 in GBM biology. This metabolic reprogramming, together with PKM2 upregulation, has been identified as a key factor in GBM prognosis and therapeutic response 51. Specifically, PKM2 was shown to induce cell-renewal and resistance to temozolomide (TMZ) treatment in GBM cells 19. In agreement with this, clinical data indicate that GBM patients with high PKM2 levels display a lower overall survival in response to radiotherapy 52. Further investigations revealed PKM2-induced radio resistance was due to reduced PKM2 enzymatic/metabolic activity, which enhanced lactate generation and derived glycolytic metabolites to the pentose phosphate pathway rather than to the TCA cycle. This effect could be counteracted by increasing PKM2 enzymatic activity inducing the formation of an active PKM2 tetramer, thereby lowering lactate and pentose phosphate pathway flux 53.

In addition to the enhanced glycolytic activity detected in GBM cells with C3G downregulation or absence, our results indicate that mitochondrial activity was also increased, particularly in U87 cells. Consequently, maximal mitochondrial respiratory capacity was significantly higher in C3G-silenced and -knockout U87 cells. However, in 12Φ12 cells non-mitochondrial respiratory capacity tended to increase upon C3G silencing, while maximal mitochondrial respiratory capacity remained unchanged (Figure 3). These data suggest that mitochondria might be more relevant for U87 than for 12Φ12 cells, which is supported by the higher mitochondria content of these cells compared to 12Φ12 cells. Therefore, 12Φ12 cells would be more dependent on glycolysis and lactate generation. This differential behavior is consistent with the metabolic heterogeneity and plasticity described in GBM cells 54 and cancer stem cells, including GBM stem-like cells 55. Indeed, GSCs have been seen to adopt distinct energetic phenotypes, either glycolytic or oxidative phosphorylation-dependent, both of which are able to sustain tumor progression 56. Moreover, the more differentiated state of U87 cells could also contribute to the observed energetic differences between U87 and 12Φ12 cells. Importantly, cells relying on oxidative phosphorylation can also switch to a glycolytic-predominant metabolism in response to metabolic stress, further supporting the high metabolic plasticity of GBM cells.

Another difference between U87 and 12Φ12 cells concerns TCA cycle metabolites. Although citrate concentration was significantly decreased in C3G-silenced U87 cells, no changes were detected in 12Φ12 cells. This discrepancy could be due to genetic or intrinsic differences between the two cell lines, as metabolic heterogeneity is a well-established feature of GBM cells. In fact, TCA cycle intermediates, including citrate, not only fuel mitochondrial respiration but also support biosynthetic and epigenetic processes, and regulate glycolytic activity, processes which could be differentially addressed in each cell model 54. Indeed, C3G-silenced U87 cells could derive citrate to fatty acid synthesis, owing to their higher levels of FAS and active ACC form. Glutamine through its conversion to a-Ketoglutarate could contribute to feed TCA cycle 57.

Beyond its glycolytic function, dimeric PKM2 also acts as a regulator of gene expression upon nuclear translocation 58,59. Once in the nucleus, PKM2 promotes the transcription of several genes, including Fatty acid synthase (*FASN*), as well as genes that reinforce the Warburg effect such as *LDHA*, *GLUT1* or *PDK1*, in specific tumor contexts 60,61. In addition, PKM2 can induce the expression of stemness-related genes through different mechanisms, including activation of EGFR signaling, which drives *KLF4* or *ALDH* expression, or by direct interaction with OCT4, which promotes the transcription of additional stemness regulators 62,63. Therefore, nuclear PKM2 could contribute to increase the expression of *FASN, LDHA* and stemness genes in C3G-silenced GBM cells, especially in 12Φ12 cells. Importantly, lactate contributes as well to the pro-tumorigenic effects of PKM2 in GBM not only within GBM cells but also by modulating the tumor microenvironment 64. In this context, lactate secreted by patient-derived GSCs and microglia/macrophages promotes histone acetylation, thereby activating immunosuppressive transcriptional programs that contribute to tumor immune evasion 65. Similarly, lactate has been reported to prevent differentiation of cancer stem cells (CSC) from intestine tumors, leading to a more proliferative CSC state through histone acetylation and the subsequent MYC activation 66. This raises the possibility that enhanced production of lactate by GBM cells with low C3G levels could also be involved in reprogramming the tumor microenvironment, which will be the focus of future investigations.

Taken together, these data show the different functions of PKM2 and lactate in regulating GBM growth, progression and treatment response. According to our results, C3G rewires GBM metabolism through PKM2, and PKM2 actions are tightly linked to C3G protein levels although specific genetic contexts must also be considered, adding an additional layer of complexity to this regulatory network.

Consistent with the essential role of PKM2 in regulating stemness, we found that C3G silencing promoted a shift towards a stem-like phenotype. Specifically, C3G-silenced or deficient cells formed a higher number of spheres, although with reduced area and cellular density, which displayed increased expression of stemness markers such as *POU5F1*, *SOX2* or *NANOG* mRNAs and Nestin and SOX2 proteins. Besides, the protein levels of GFAP, a marker of differentiated astrocytes, decreased in C3G knockout U87 cells. Functionally, these changes translated into a higher tumor-initiating capacity as determined by LDA. In line with these findings, C3G knockout has been previously reported to impair the differentiation of embryonic stem cells 67, and its downregulation also promoted the expression of stemness markers such as CD133 and CD44 in liver progenitor cells 32.

Given the central role of PKM2 in the control of GBM metabolism and stemness, pharmacological strategies targeting glycolysis and specifically PKM2, have been explored in several preclinical studies. One of those approaches involves the use of compound 3K, a recently described small molecule that specifically inhibits PKM2 metabolic activity by stabilizing an inactive tetrameric form in prostate cancer cells 65. Compound 3K also induces apoptosis and autophagy through AMPK activation and inhibition of Akt and mTOR in ovarian cancer 68,69. Our findings show that PKM2 inhibition with compound 3K also promotes cell death in both adherent GBM cells and GBM neurospheres, with apoptosis being the main mechanism. Interestingly, C3G downregulation reduces the sensitivity of adherent cells to PKM2 inhibition, while C3G absence has an opposite effect. This points out the requirement of a minimum level of C3G to improve cell viability upon PKM2 inhibition, which is consistent with the increased survival of mice bearing a hypomorphic C3G allele 30 compared to C3G knockout mice 70. On the other hand, PKM2 inhibition impaired sphere formation in C3G-silenced 12Φ12 cells, diminished its initiating capacity and reduced the expression of stem-related genes, underscoring a PKM2-dependent enhancement of stemness in GBM cells with low levels of C3G. Therefore, PKM2 inhibition will selectively target GSCs with low levels of C3G, decreasing its tumor initiating capacity and promoting their death. Nevertheless, specific subtypes of GSCs could be more sensitive based on their genetic characteristics.

In addition, in this study we have identified a mechanism that might be responsible for PKM2 upregulation upon C3G silencing or knockout in GBM cells. We show that C3G regulates *PKM* alternative splicing through modulation of the expression of splicing factors. Specifically, C3G downregulation increases the expression of PTBP1 in GBM cells through an ERKs-dependent mechanism, which in turn promotes *PKM* splicing towards PKM2 expression (Figure 4), consistent with the well-established role of PTBP1 in this process 71,72 and with our results upon PTBP1 silencing. Furthermore, PTBP1 knockdown in C3G-silenced GBM cells led to reduced expression of mRNAs encoding additional splicing factors, including SRSF2, SRSF3, hnRNPA1 and hnRNPA2/B1, all of which are known regulators of *PKM* splicing 60,73,74,75,76,77. These splicing factors may therefore cooperate with PTBP1 to sustain PKM2 expression in GBM cells with low C3G levels, with a preferential role for SRSF3, whose protein levels increased in a PTBP1-dependent manner. C3G has previously been shown to regulate splicing in muscle cell lines through the control of splicing factors such as SRSF2 and by additional mechanisms 47. Moreover, in a hepatocellular carcinoma cell line, C3G silencing has been reported to upregulate PKM2 in a PTBP1-dependent manner 38. Here, we extend this role to GBM by uncovering PTBP1 as a splicing factor whose expression is regulated by C3G (likely through GEF-dependent and independent mechanisms), which in turn controls *PKM* splicing, thereby linking C3G to GBM cell metabolism and stemness. Moreover, PTBP1-mediated regulation of SRSF3 expression would contribute to promoting PKM2 expression, revealing previously unrecognized mechanisms.

Although we have demonstrated here that PTBP1-PKM2 axis mediates, at least in part, the enhanced GBM stemness and tumor initiating capacity induced by C3G knockdown or knockout, we cannot exclude PKM2-independent effects of PTBP1, SRSF3 and other splicing factors 78. Indeed, dysregulation of the splicing machinery, including PTBP1 and SRSF3 upregulation, has been shown to promote GBM growth, aggressiveness and progression 79. Our data support a similar effect of PTBP1 silencing and PKM2 (chemical or genetic) inhibition, underscoring the relevance of PTBP1-PKM2 to regulate GBM stemness.

In conclusion, our study reveals novel mechanisms by which C3G regulates tumorigenesis in GBM, with PKM2 playing a pivotal role. We showed that C3G downregulation enhances PKM2 expression through modulation of splicing, thereby promoting stemness and tumor initiating capacity in all GBM cells used in this study (see graphical abstract in Figure S11). Importantly, despite differences at the level of specific TCA cycle metabolites and mitochondrial respiration between U87 and 12Ф12 cells, glycolysis and PKM2 are similarly regulated by C3G in both cell lines (likely through GEF-dependent and independent mechanisms), as well as in HCO1 cells, underlining the robustness and biological relevance of this mechanism.

These findings not only uncover a new regulatory pathway modulating GBM progression but could also have implications for GBM diagnosis, prognosis and therapeutic intervention in specific GBM patient subgroups with different molecular characteristics in the future. However, more studies are needed to determine the potentiality of stratifying GBM patients with low C3G and high PKM2 levels within the different GBM subtypes (i.e. neural, pro-neural, mesenchymal and classical) 80 to improve prognosis and/or treatment. Nevertheless, patient treatment with PKM2 inhibitors represents a big challenge as they need to be able to enter in the brain through blood-brain barrier.

## Supplementary Material

Supplementary methods, figures and tables.
