# Supplementary material for: C3G Downregulation Enhances Stemness in Glioblastoma Cells by Promoting PKM2 Upregulation
Source: Int J Biol Sci. 2026 Jul 1;22(12):6500–20. doi: 10.7150/ijbs.126349 (PMC13412097; doi:10.7150/ijbs.126349)
Supplement: Supplementary file 1 — Supplementary methods, figures and tables. [file ijbsv22p6500s1.pdf]

SUPPLEMENTARY INFORMATION

SUPPLEMENTARY MATERIAL AND METHODS

Primers used for RT-qPCR analysis

Primers used for:

PTBP1 f- GTGACTACACACGCCCAGAC and r- TATACCAGGTGCACCGAAGG; *PKM1* f- GAGCATGATCAAGAAGCCCC and r- GGCTCGCACAAGTTCTTCAA; *PKM2 f-* GAGCATGATCAAGAAGCCCC and r- AAGTGGTAGATGGCAGCCTC; *SRSF2* f- CGTGCCTGAAACTGAAACCA and r- TCCCCAAGTCCTCCGTTTAC; *SRSF3* f- CCGGGTGAGTGAGAGAGTTG and r- AATCCACACGCTCCGATGAG; *hnRNPA1* f- AAGCAATTTTGGAGGTGGTG and r- ATAGCCACCTTGGTTTCGTG; *hnRNPA2/B1* f- GACTGTGTGGTAATGAGGGATCCT and r- GCTCAACTACTCTCCCATCAATTGA; *POU5F1* f- TGCTCCAGCTTCTCCTTCTC and r- GTGGAGAGCAACTCCGATG; *SOX2* f- CCAGCAGACTTCACATGTCC and r- ACATGTGTGAGAGGGGCAGT; *NANOG* f- TTTTTGCGACAGTCTTCTCTGC and r- ATTCAGGACAGCCCTGATTCTTC; *BMI1* f- AATCCCCACCTGATGTGTGT and r- GCTGGTCTCCAGGTAACGAA, *PROM1* f- ATGGCAACAGCGATCAAGG and r- GTACTTTGTTGGTGCAAGCTCT; *CD44* f- AAGGTGGAGCAAACACAACC and r- TCGACTGTTGACTGCAATGC ; and *TBP* f- GGCTGTTTAACTTCGCTTCCG and r- CAAGAAACAGTGATGCTGGGTC.

Additional antibodies

Antibodies against ACC (Cell signaling 3676S), P-ACC (Cell signaling 3661), FAS (Cell signaling 3189), CPT1A (Proteintech 15184-1-AP), P-Thr202/Y204-ERKs (Cell signaling 9101), ERKs (Cell signaling 9102), and SRSF3 (SelleckChem F3211).

HCO1 cell culture

The non-commercial GBM HCO1 cell line with a stem-like phenotype, derived from a patient GBM tumor (mycoplasma free), was used for key experiments. C3G-silenced cells were previously generated in the laboratory using lentiviral particles with shRNAs against human C3G (Manzano et al. 2021, Cell Death Dis. 12(4):348).
Cells were grown in DMEM supplemented with 10% fetal bovine serum (FBS) under attachment conditions for 2D assays.

GBM neurospheres were generated as described in the main text for U87 and 12Φ12 cells.

Inhibition of ERKs

Non-silenced and C3G-silenced 12Φ12 GBM cells were treated with the MEK inhibitor PD98059 (10 μM) for 48 hours to inhibit ERKs activation.

Generation of spheres in a medium without pyruvate

Spheres were generated in the sphere medium as described in the main text, with the only difference that DMEM:Ham's F-12 medium (Gibco 11039021) lacked 0.5 mM pyruvate.

C3G rescue experiments

U87C3GKO cells were transfected with 1 ug of empty PLTR2 plasmid (control) and plasmids containing either full-length C3G (PLTR2-C3GFL, 2 μg DNA) or a catalytic domain-lacking C3G mutant (PLTR2-C3GΔCat, 1 μg of DNA) using Lipofectamine™

3000 (Invitrogen #L3000015) according to the manufacturer's instructions. Experiments were performed 72h-96 h post-transfection.

CellTrace *in vitro* assay

shControl and shC3G 12Φ12 cells dissociated as single cells were labelled with the CellTrace Violet (CTV) fluorescent dye (Invitrogen C34571) according to the manufacturer's protocol. Then, 5.5 × 10^5 12Φ12 cells were seeded onto 60-mm non-adherent dishes in neurosphere medium. Every 24 hours, representative images were acquired using a Nikon Eclipse TE300 microscope coupled to a camera to track CTV-positive cells. After culturing for 2 or 7 days, cells were dissociated into single-cell suspensions and analyzed by flow cytometry (FACSCalibur™). Data was analyzed with FlowJo (Tree Star) to quantify the percentage of CTV-positive cells.

Analysis of mitochondria content by flow cytometry

Mitochondrial content was assessed in live cells by flow cytometry using MitoTracker Deep Red 633 (Invitrogen M22426). Cells at 60-70% confluency were incubated with MitoTracker 10 nM diluted in complete medium for 30 minutes at 37ºC. Subsequently, cells were harvested, washed once with PBS and analyzed using a FACSymphony A1 flow cytometer (BD Biosciences, San Jose, CA, USA).

Viability assays in non-tumor cells

Cell viability in response to compound 3K (SelleckChem S8616) was assessed by crystal violet staining. Human lung fibroblasts CF5 were kindly donated by Dr. Alcaraz (University of Barcelona, Spain) (Ikemori et al. 2020, Cancer Res. 2020 80(2):276-290), were seeded in a 24-well plate at a density of 3 × 10^5 per well and incubated for 24h, followed by treatment with compound 3K (2.5 and 5 μM) for 24, 48, and 72h. Cells were subsequently stained with 0.05% crystal violet (Panreac 251762.1606) to evaluate viability.

Analysis of apoptosis by flow cytometry

The analysis of cell cycle in U87 cells was performed in a FACScalibur flow cytometer (BD Biosciences, San Jose, CA, USA). Cells were fixed with ethanol at –20ºC (2min), washed with PBS and treated with RNase A for 30min at 37ºC. Then, DNA was stained with propidium iodide (PI). Measurements were carried out using a Double Discriminator Module to discriminate doublets. 10.000 cells were acquired per sample. The percentage of cells in subG0/1, with DNA content lower than 2C, were considered apoptotic and calculated using Multicycle software (Phoenix software).

Analysis of active caspase 3 by immunofluorescence

Active caspase 3 was analyzed by immunofluorescence using anti-cleaved caspase 3 antibody (CC3 (Cell signaling 9661S)) in 12Φ12 cells. Fluorescence intensity was measured in at least 8 fields per condition and represented by the Integrated Density (ID) parameter (ID = Mean value of fluorescence intensity x positive area percentage) referred to DAPI area.

Analysis of subcellular localization of PKM2 by confocal microscopy

PKM2 immunostaining was performed as described in the main text using PKM2 (Cell Signaling 3198S) and DAPI staining Images were acquired using an FV1200

confocal microscope (Olympus, Tokyo, Japan). Nuclear and total PKM2 fluorescence intensities were quantified using ImageJ software (v1.52a, National Institutes of Health, USA). Images were analyzed by measuring the corrected total cell fluorescence (CTCF). Briefly, regions of interest corresponding to the nucleus or the whole cell were manually defined using the freehand selection tool. Background fluorescence was determined by performing three independent measurements in cell-free areas of each image. CTCF values were calculated according to the following formula:

$$CTCF = \text{Integrated Density} - (\text{Area of selected region} \times \overline{\text{background fluorescence}})$$

The resulting values were used to quantify total cellular and nuclear PKM2 fluorescence intensity, as well as nuclear-to-cytoplasmic PKM2 ratio.

F-actin staining was also performed using rhodamine-conjugated phalloidin (Sigma Aldrich#P1951) as described (Manzano et al. 2021, Cell Death Dis. 12(4):348).

SUPPLEMENTARY TABLES AND FIGURES

Supplementary Table 1 – Results from proteome-wide analysis: proteins over-represented in parental U87 cells compared to U87shC3G cells

ID (gene)	Protein name	Score	Matches (peptides)	Fold change(*)	*p* value
ACTB	Actin, cytoplasmic 1	228	24	1.3	**
ACTB	Actin, cytoplasmic 1	177	15	1.2	**
ACTA	Actin, aortic smooth muscle	175	14	1.3	**
POTEE	POTE ankyrin domain family member E	109	9	1.3	**
AHSA1	Activator of 90 kDa heat shock protein ATPase homolog 1	100	6	1.2	**
POTEE	POTE ankyrin domain family member E	89	6	1.2	**
RSSA	40S ribosomal protein SA	89	6	1.2	**
ACTBL	Beta-actin-like protein 2	79	6	1.3	**
CATD	Cathepsin D	79	6	1.3	**
IF4H	Eukaryotic translation initiation factor 4H	62	3	1.5	*
ACTBL	Beta-actin-like protein 2	51	8	1.2	**
TADBP	TAR DNA-binding protein 43	46	1	1.3	**
PGK1	Phosphoglycerate kinase 1	44	3	1.3	**
TBCA	Tubulin-specific chaperone A	43	3	1.3	**
COTL1	Coactosin-like protein	41	1	1.3	**
EFTU	Elongation factor Tu, mitochondrial	41	2	1.3	**

EIF3G	Eukaryotic translation initiation factor 3 subunit G	41	3	1.3	**
NFH	Neurofilament heavy polypeptide	38	3	1.5	*
ASAP2	Arf-GAP with SH3 domain, ANK repeat and PH domain-containing protein 2	37	1	1.3	**
GLP2R	Glucagon-like peptide 2 receptor	37	3	1.2	**
ENOA	Alpha-enolase	36	2	1.3	**
MED16	Mediator of RNA polymerase II transcription subunit 16	36	1	1.3	**
CXCL5	C-X-C motif chemokine 5	35	1	1.5	*
CENPF	Centromere protein F	34	1	1.3	**
PEX1	Peroxisome biogenesis factor 1	31	3	1.5	*
BD1L1	Biorientation of chromosomes in cell division protein 1-like 1	29	3	1.5	*
BD1L1	Biorientation of chromosomes in cell division protein 1-like 1	28	3	1.2	**
CATD	Cathepsin D OS=Homo sapiens	28	2	1.2	**
O2T35	Olfactory receptor 2T35	28	2	1.3	**
4ET	Eukaryotic translation initiation factor 4E transporter	27	1	1.5	*
AL4A1	Delta-1-pyrroline-5-carboxylate dehydrogenase, mitochondrial	27	1	1.5	*
HCD2	3-hydroxyacyl-CoA dehydrogenase type-2	26	4	1.5	*
SI1L3	Signal-induced proliferation-associated 1-like protein 3	26	1	1.5	*
SOX5	Transcription factor SOX-5	26	1	1.5	*
AT1A4	Sodium/potassium-transporting ATPase subunit alpha-4	22	2	1.2	**
PTPRQ	Phosphatidylinositol phosphatase PTPRQ	20	1	1.2	**
RHBT3	Rho-related BTB domain-containing protein 3	20	1	1.3	**
MIER1	Mesoderm induction early response protein 1	17	1	1.5	*
OCTC	Peroxisomal carnitine O-octanoyltransferase	16	1	1.2	**

RBM46	Probable RNA-binding protein 46	16	1	1.2	**
YD286	Glutaredoxin-like protein C5orf63	16	1	1.2	**
AGRF5	Adhesion G protein-coupled receptor F5	14	1	1.2	**
LC7L2	Putative RNA-binding protein Luc7-like 2	14	1	1.2	**
LIPB1	Liprin-beta-1	14	1	1.2	**
SDHF2	Succinate dehydrogenase assembly factor 2, mitochondrial	14	1	1.2	**
VATD	V-type proton ATPase subunit D	14	1	1.2	**

***(*) Fold change:** levels detected in parental U87 cells vs. levels in U87shC3G.* (n=4).

<u>Supplementary Table 2</u> – Results from proteome-wide analysis: proteins over-represented in U87shC3G cells compared to parental U87 cells. Pyruvate kinase protein is highlighted in red.

ID (gene)	Protein name	Score	Matches (peptides)	Fold change(*)	*p* value
LMNA	Prelamin-A/C	213	22	1.4	*
PCBP1	Poly(rC)-binding protein 1	192	14	1.3	*
STIP1	Stress-induced-phosphoprotein 1	171	19	1.4	*
ALDOA	Fructose-bisphosphate aldolase A	147	9	1.3	*
CAPG	Macrophage-capping protein	132	7	1.4	*
TPIS	Triosephosphate isomerase	126	7	1.2	**
ACTB	Actin, cytoplasmic 1	125	7	1.3	*
PGK1	Phosphoglycerate kinase 1	110	20	1.3	*
POTEE	POTE ankyrin domain family member E	83	4	1.3	*
POTEI	POTE ankyrin domain family member I	74	3	1.3	*
ALDOA	Fructose-bisphosphate aldolase A	64	3	1.4	*
GALK1	Galactokinase	54	1	1.4	*
PRS10	26S protease regulatory subunit 10B	46	4	1.3	*
AIFM1	Apoptosis-inducing factor 1, mitochondrial	43	2	1.4	*

ASAP2	Arf-GAP with SH3 domain, ANK repeat and PH domain-containing protein 2	42	2	2.1	**
HNRDL	Heterogeneous nuclear ribonucleoprotein D-like	42	2	1.4	*
HNRPD	Heterogeneous nuclear ribonucleoprotein D0	42	2	1.4	*
CC181	Coiled-coil domain-containing protein 181	41	1	1.3	*
CTCFL	Transcriptional repressor CTCFL	41	1	1.3	*
ENOA	Alpha-enolase	41	1	1.3	*
ISK5	Serine protease inhibitor Kazal-type 5	41	1	1.3	*
PERI	Peripherin	41	1	1.3	*
KPYM	**Pyruvate kinase PKM**	40	3	1.4	*
HSPB1	Heat shock protein beta-1	37	3	1.2	**
SNED1	Sushi, nidogen and EGF-like domain-containing protein 1	37	1	2.1	**
ANKY2	Ankyrin repeat and MYND domain-containing protein 2	36	2	1.4	*
MAK16	Protein MAK16 homolog	36	2	1.4	*
AIFM1	Apoptosis-inducing factor 1, mitochondrial	35	1	1.3	*
ALDOA	Fructose-bisphosphate aldolase A	35	2	1.3	*
FMN1	Formin-1	35	1	1.3	*
MA7D3	MAP7 domain-containing protein 3	34	2	1.3	*
ACTBL	Beta-actin-like protein 2 OS=Homo sapiens GN=ACTBL2 PE=1 SV=2	33	3	1.3	*
QRIC2	Glutamine-rich protein 2	33	1	2.1	**
ERI1	3'-5' exoribonuclease 1	31	1	1.4	*
CCDC6	Coiled-coil domain-containing protein 6	30	1	1.4	*
PSD13	26S proteasome non-ATPase regulatory subunit 13	30	1	1.4	*
QCR2	Cytochrome b-c1 complex subunit 2, mitochondrial	30	2	1.3	*
CRK	Adapter molecule crk	29	1	2.1	**
PPR36	Protein phosphatase 1 regulatory subunit 36	29	1	2.1	**
RGSL	Regulator of G-protein signalling protein-like	29	1	1.3	*

RGSL	Regulator of G-protein signalling protein-like	29	1	1.4	*
TERA	Transitional endoplasmic reticulum ATPase	29	1	1.4	*
ALDOC	Fructose-bisphosphate aldolase C	28	2	1.3	*
APOP1	Apoptogenic protein 1, mitochondrial	28	1	1.3	*
ENOA	Alpha-enolase	28	3	1.3	*
RAD50	DNA repair protein RAD50	28	2	1.3	*
FANCB	Fanconi anemia group B protein	27	1	1.3	*
SPIN3	Spindlin-3	27	1	1.3	*
DOCK6	Dedicator of cytokinesis protein 6	24	1	1.2	**
ARI4B	AT-rich interactive domain-containing protein 4B	23	5	1.3	*
DDX53	Probable ATP-dependent RNA helicase DDX53	23	1	1.3	*
FACR1	Fatty acyl-CoA reductase 1	23	1	1.3	*
GSTP1	Glutathione S-transferase P	23	1	2.1	**
NSF	Vesicle-fusing ATPase	23	1	1.3	*
PLXC1	Plexin-C1	23	1	1.3	*
RFIP3	Rab11 family-interacting protein 3	23	2	1.2	**
RFIP3	Rab11 family-interacting protein 3	23	1	1.3	*
RFIP3	Rab11 family-interacting protein 3	21	3	2.1	**
RIOK3	Serine/threonine-protein kinase RIO3	21	1	1.2	**
AT1A4	Sodium/potassium-transporting ATPase subunit alpha-4	20	2	1.4	*
CTRO	Citron Rho-interacting kinase	20	2	1.3	*
ERO1A	ERO1-like protein alpha	20	1	1.3	*
KCC4	Calcium/calmodulin-dependent protein kinase type IV	20	1	2.1	**
Z518B	Zinc finger protein 518B	20	2	2.1	**
B3GA2	Galactosylgalactosylxylosyl protein 3-beta-glucuronosyltransferase 2	19	1	2.1	**
DEN1C	DENN domain-containing protein 1C	19	1	1.3	*
CO6A3	Collagen alpha-3(VI) chain	18	2	1.4	*

JCAD	Junctional protein associated with coronary artery disease	17	1	1.2	**
C102A	Coiled-coil domain-containing protein 102A O	16	1	1.2	**
MINT	Msx2-interacting protein	16	3	1.4	*

(*) Fold change: *levels detected in U87shC3G cells vs. levels in parental U87 cells.* (n=4).

SUPPLEMENTARY FIGURES

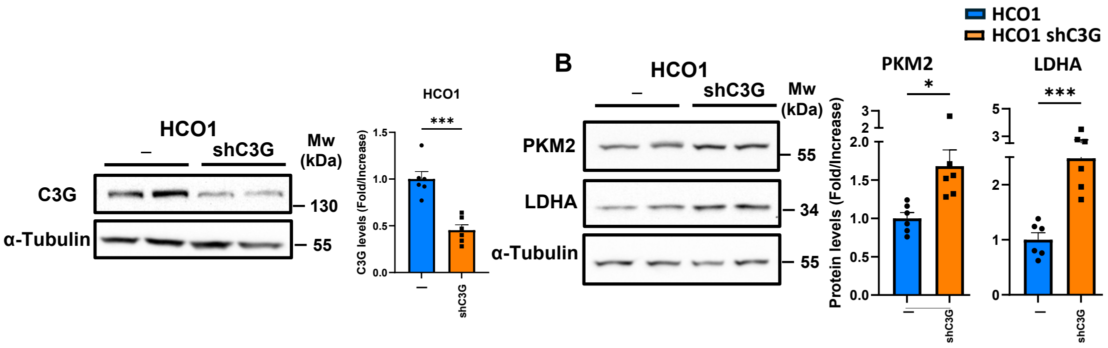

Figure S1-PKM2 and LDHA are upregulated in C3G-silenced GBM HCO1 cells. Non-silenced and C3G-silenced HCO1 were used. **A)** Western blot analysis of C3G protein levels normalized with α-Tubulin (left panel). The histogram shows the quantification (main value ± S.E.M.) of the ratio C3G/α-Tubulin of different western blots (right panel) (n=6). **B)** Western blot analysis of PKM2 and LDHA protein levels normalized with α-Tubulin (left panel). Histograms show the quantification (main value ± S.E.M.) of the ratio PKM2/α-Tubulin and LDHA/α-Tubulin of different western blots (n=6). *p≤0.05 and ***p≤0.001 compared to non-silenced cells.

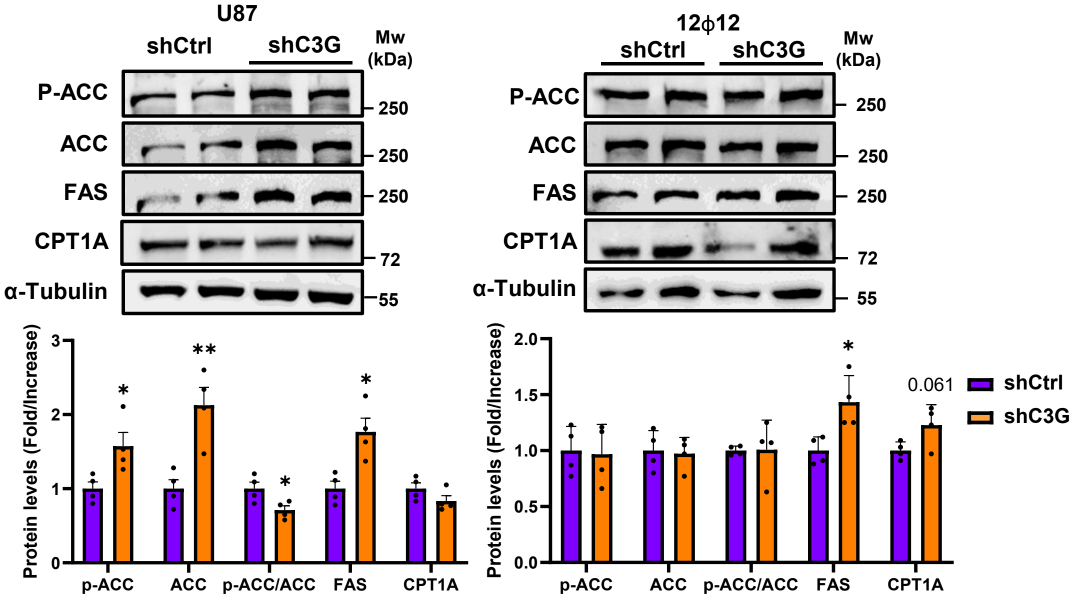

Figure S2-Effect of C3G silencing on the expression of enzymes from fatty acid metabolism in GBM cells. Non-silenced (shControl) and C3G-silenced (shC3G) U87 and 12Φ12 were used. Top panels, western blot analysis of ACC, P-ACC, FAS and CPT1A protein levels normalized with α-Tubulin. Lower panels, histograms showing the quantification (main value ± S.E.M.) of the ratio ACC/α-Tubulin, P-ACC/α-Tubulin, FAS/α-Tubulin and CPT1A/α-Tubulin of different western blots (n=4). *p≤0.05 and **p≤0.01 compared to non-silenced cells.

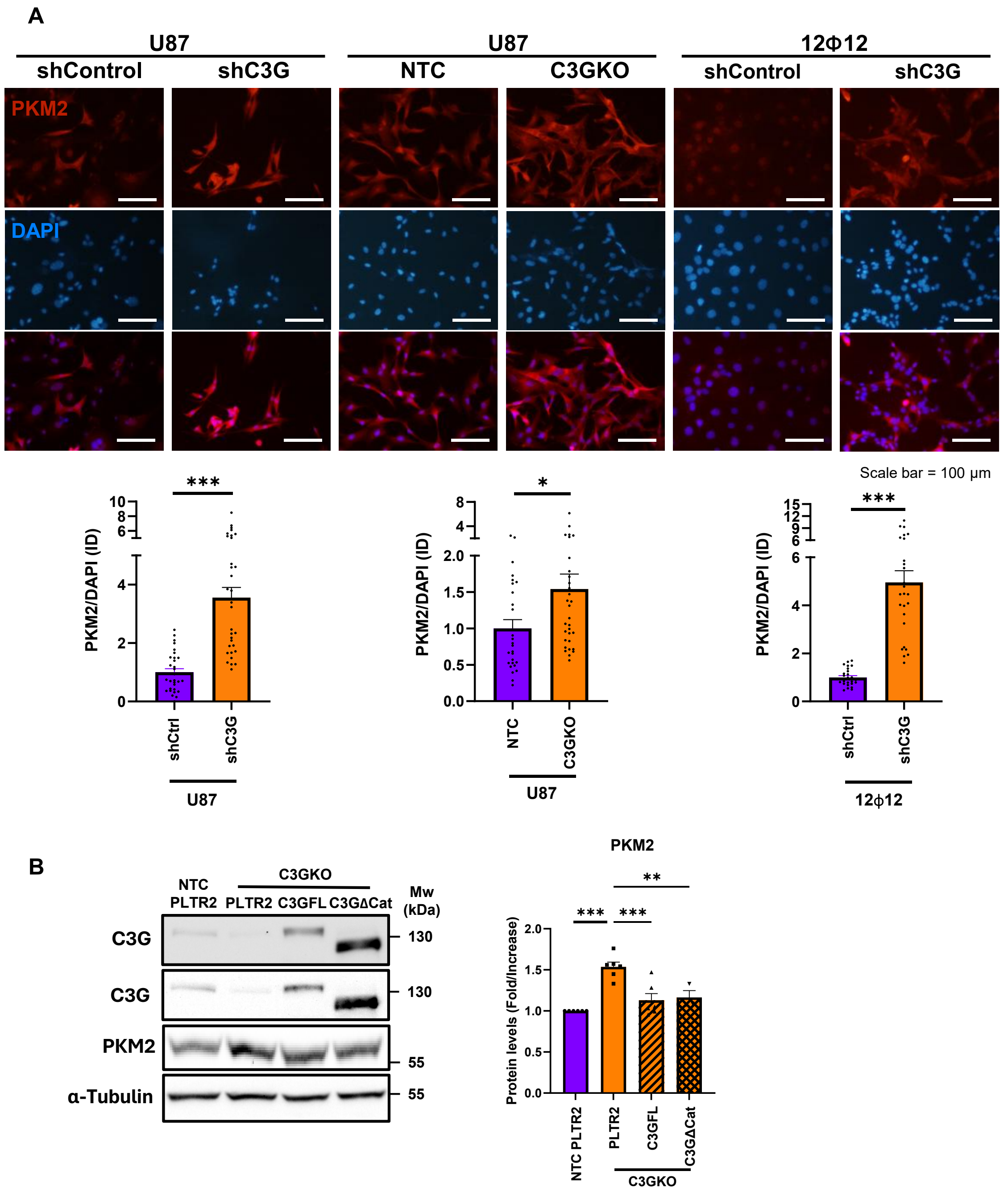

Figure S3-PKM2 is upregulated in C3G-silenced and knockout GBM cells. Effect of C3G rescue in C3GKO GBM cells. A) Immunofluorescence analysis of PKM2 in C3G-silenced (U87shC3G and 12Φ12shC3G) and their control cells (U87shControl (U87shCtrl) and 12Φ12shControl (12Φ12shCtrl)), and in C3G knockout (U87C3GKO) and its control (U87NTC). PKM2, DAPI and merge representative images are shown.

Histograms show fluorescence intensity of PKM2 versus DAPI. *p≤0.05 and ***p≤0.001 compared to non-silenced cells (n=3). Scale bars (50 μm). **B)** Western blot analysis of C3G (two different exposures) and PKM2 normalized with α-Tubulin in U87C3GKO cells transfected with the empty vector (PLTR2), a construct with C3G full length (PLTR2-C3GFL) or with C3G lacking the catalytic domain (PLTR2-C3GΔCat) and their control (NTC). Histogram shows the quantification (main value ± S.E.M.) of the ratio PKM2/α-Tubulin. **p≤0.01 and ***p≤0.001 compared as indicated (n=5).

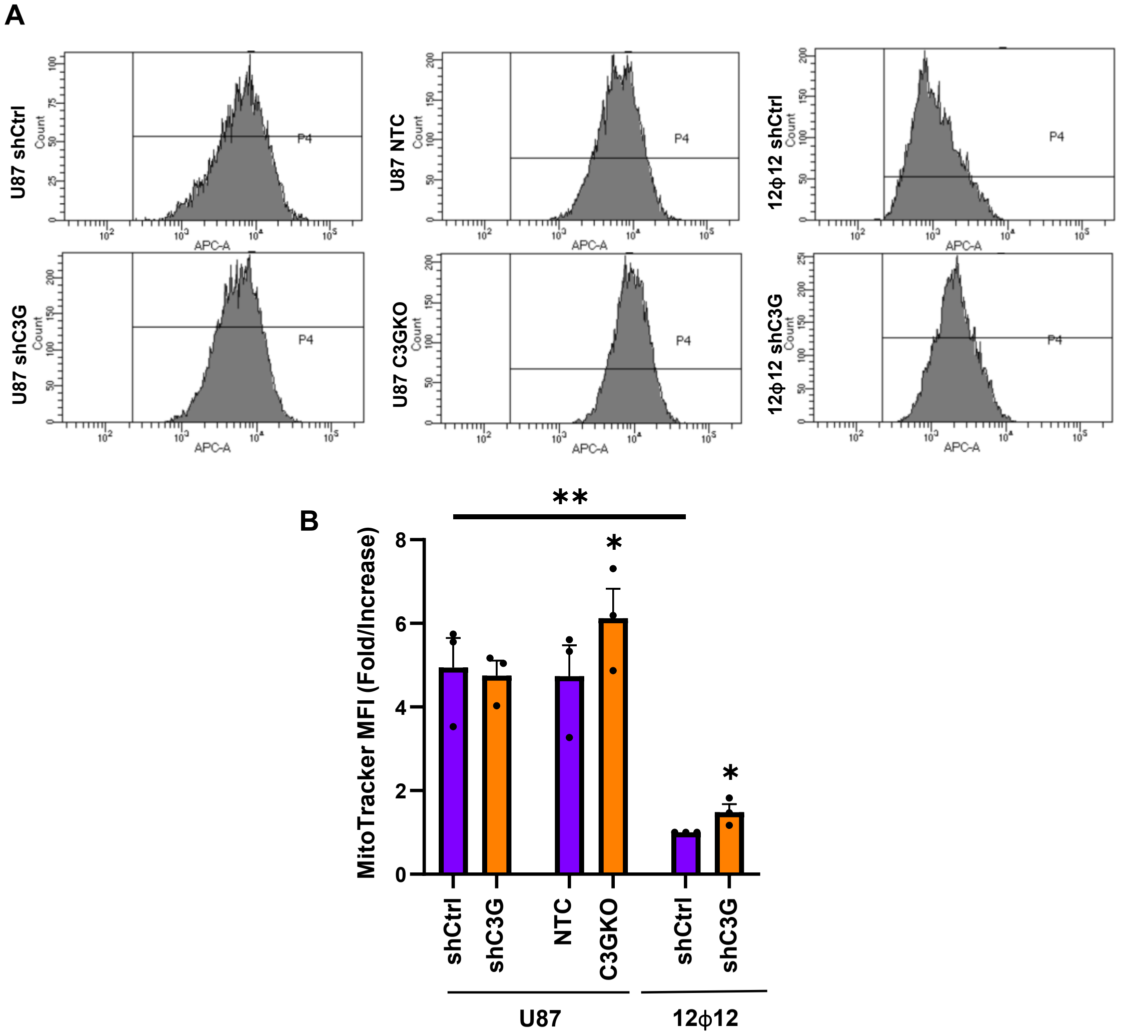

Figure S4-U87 GBM cells show a higher mitochondria mass than 12Φ12 GBM cells. Mitochondria staining with Mito Tracker Deep Red 633 in C3G-silenced (U87shC3G and 12Φ12shC3G) and their control cells (U87shControl (U87shCtrl) and 12Φ12shControl (12Φ12shCtrl)), and in C3G knockout (U87C3GKO) and its control (U87NTC). **A)** Representative flow cytometry plots representing the number of cells and the fluorescence intensity. **B)** The histogram shows the mean fluorescence intensity in the different cell lines expressed as fold increase. *p≤0.05 compared to non-silenced cells and **p≤0.01 compared as indicated (n=3).

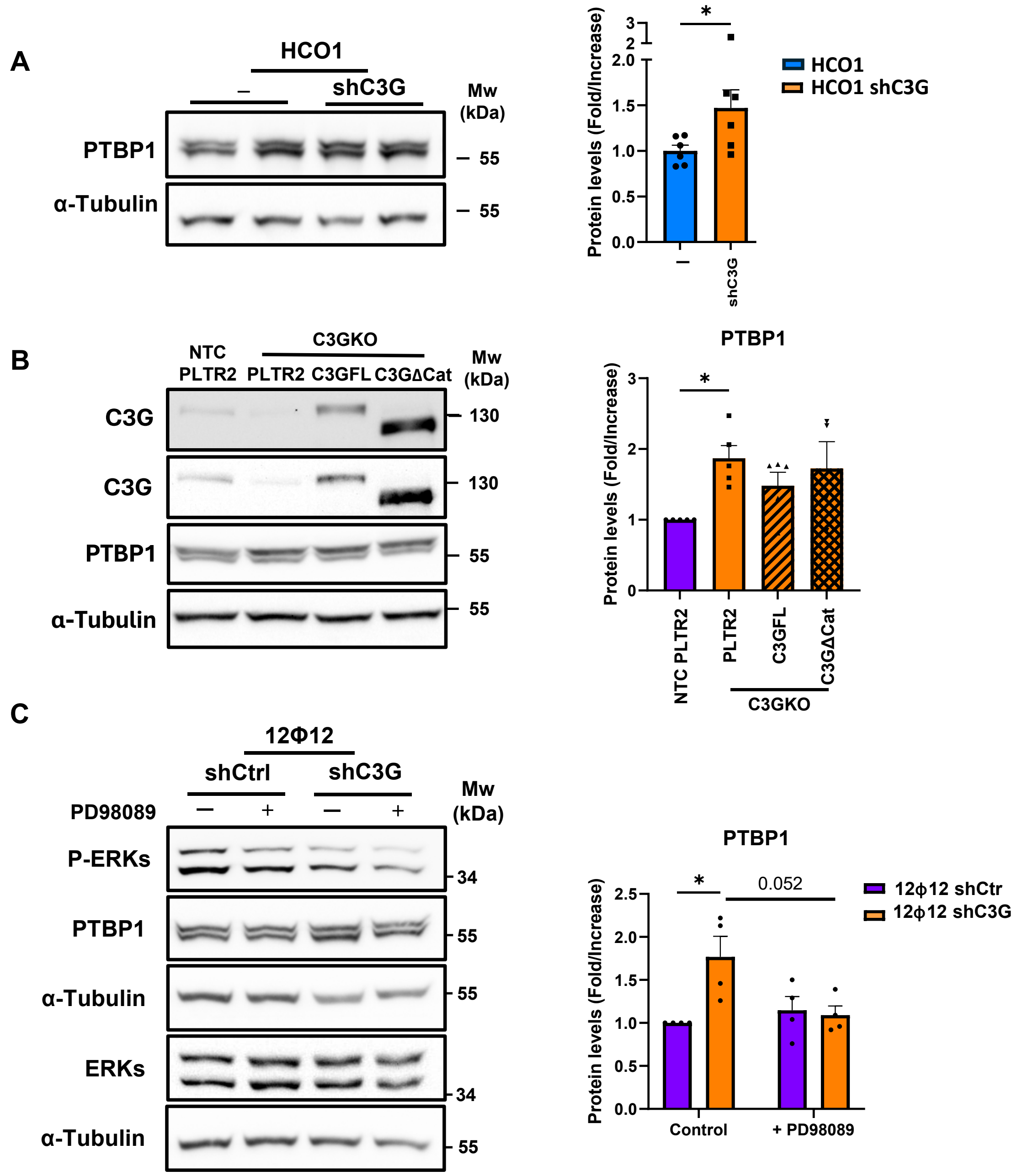

Figure S5-ERKs mediate PTBP1 upregulation induced by C3G-silencing in GBM cells. A) Western blot analysis of PTBP1 protein levels normalized with α-Tubulin (left panel) in HCO1 cells. The histogram shows the quantification (main value ± S.E.M.) of the ratio PTBP1/α-Tubulin of different western blots (right panel) (n=6). **B)** Effect of C3G re-expression in U87C3GKO cells. Western blot analysis of C3G (two different exposures) and PTBP1 normalized with α-Tubulin in U87C3GKO cells transfected with the empty vector (PLTR2), a construct with C3G full length (PLTR2-C3GFL) or with C3G lacking the catalytic domain (PLTR2-C3GΔCat) and their control (NTC). Histogram shows the quantification (main value ± S.E.M.) of the ratio PTBP1/α-Tubulin (n=5). **C)** Effect of ERKs inhibition on PTBP1 expression. Non-silenced and C3G-silenced 12Φ12 cells were treated with PD98059 (10 μM) for 48h. Then, PTBP1, P-ERKs and ERKs were analyzed by western blot normalizing with α-Tubulin. P-ERKs/α-Tubulin and PTBP1/α-Tubulin ratio was determined. Histogram shows the quantification (main value ± S.E.M.) of the ratio PTBP1/α-Tubulin. *p≤0.05 compared as indicated (n=4).

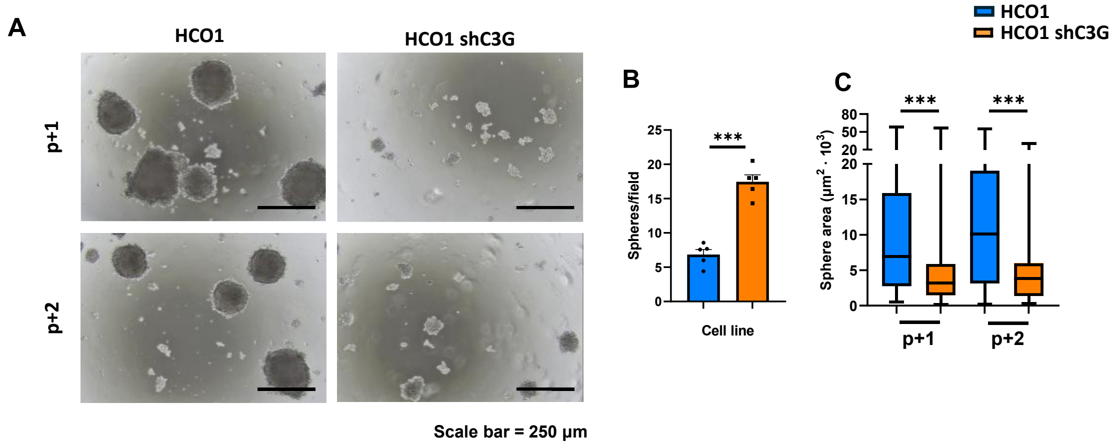

Figure S6-C3G silencing enhanced sphere-forming capacity in HCO1 GBM cells. Non-silenced and C3G-silenced HCO1 were used. **A)** Representative phase-contrast microscope images of spheres at passages 1 and 2. **B)** Histogram showing sphere number per field (n=6). **C)** Histogram showing sphere area expressed as $\mu m^2 x10^3$. ***p≤0.001 compared as indicated.

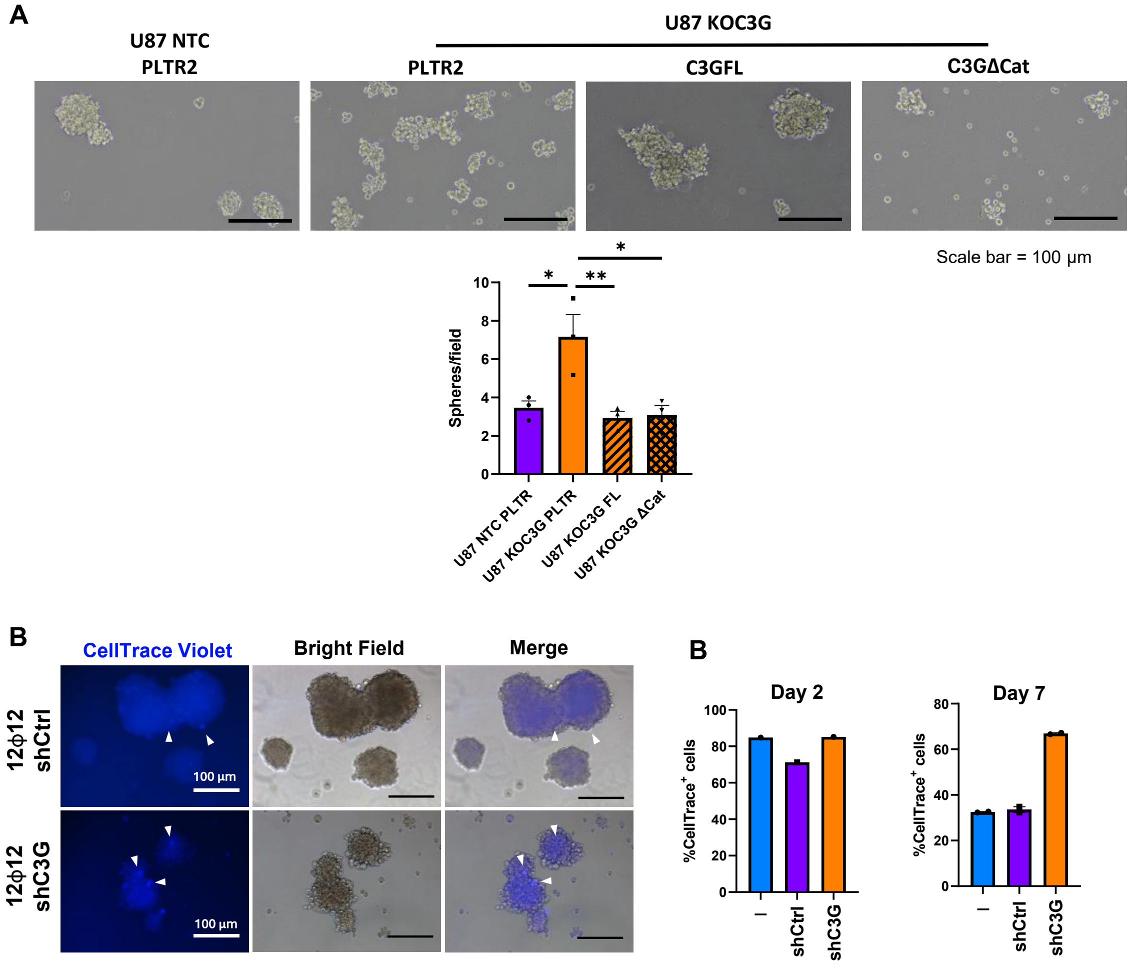

Figure S7-Effect of C3G on sphere-forming capacity and proliferation. A) Analysis of C3G rescue on sphere formation. U87C3GKO cells transfected with the empty vector (PLTR2), a construct with C3G full length (PLTR2-C3GFL) or with C3G lacking the catalytic domain (PLTR2-C3GΔCat) and their control (NTC). Representative phase-contrast microscope images of spheres at passage 1 (left panel). Histogram showing

sphere number per field (right panel) (n=3). **B)** Cell trace analysis in C3G-silenced and control 12Φ12 neurospheres using Cell Trace violet. Representative phase-contrast microscopic images of stained spheres. Scale bars (100 μm) (left panel). Histograms show the percentage of stained cells after 2 and 7 days (right panels). *p≤0.05 and **p≤0.01 compared as indicated.

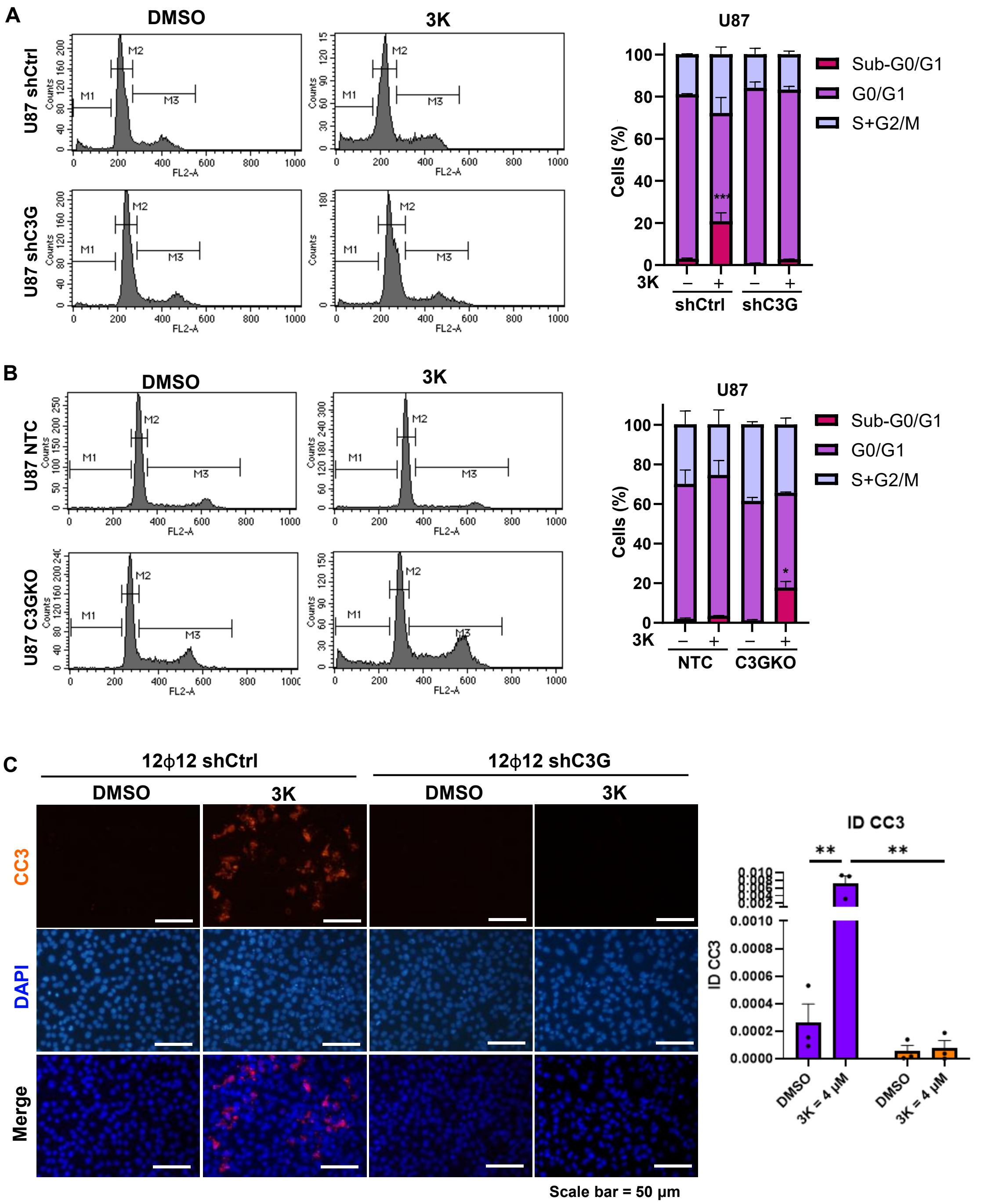

Figure S8-Inhibition of PKM2 with 3K compound induces apoptosis in C3G deficient GBM cells, while in C3G silenced cells inhibits apoptosis. A-B) Flow cytometry analysis of cell cycle in C3G-silenced (U87shC3G) and its control (U87shControl (U87shCtrl) **(A)**, and in C3G knockout (U87C3GKO) and its control (U87NTC) **(B)**. Left panels, representative plots representing the number of cells and the fluorescence intensity of PI. M1 corresponds to cells with a DNA content lower than 2C

(subG0/G1 phases), M2 to cells with 2C DNA (G0/G1 phases) and M3 cells with a DNA content higher than 2C (S+G2/M phases). Right panels, histograms showing the percentage (%) of cells in the different phases of the cell cycle with subG0/G1 corresponding to apoptotic cells (n=3). **C)** Analysis of cleaved caspase 3 (CC3) levels by immunofluorescence in C3G-silenced (12Φ12shC3G) and non-silenced (12Φ12shControl (12Φ12shCtrl)) cells. Left panels, representative fluorescence microscopy images of CC3, nuclear DAPI staining and merge. Right panel, histogram showing the intensity of CC3 staining. *p≤0.05, **p≤0.01 and ***p≤0.001 comparing cells treated with 3K with non-treated cells (n=3).

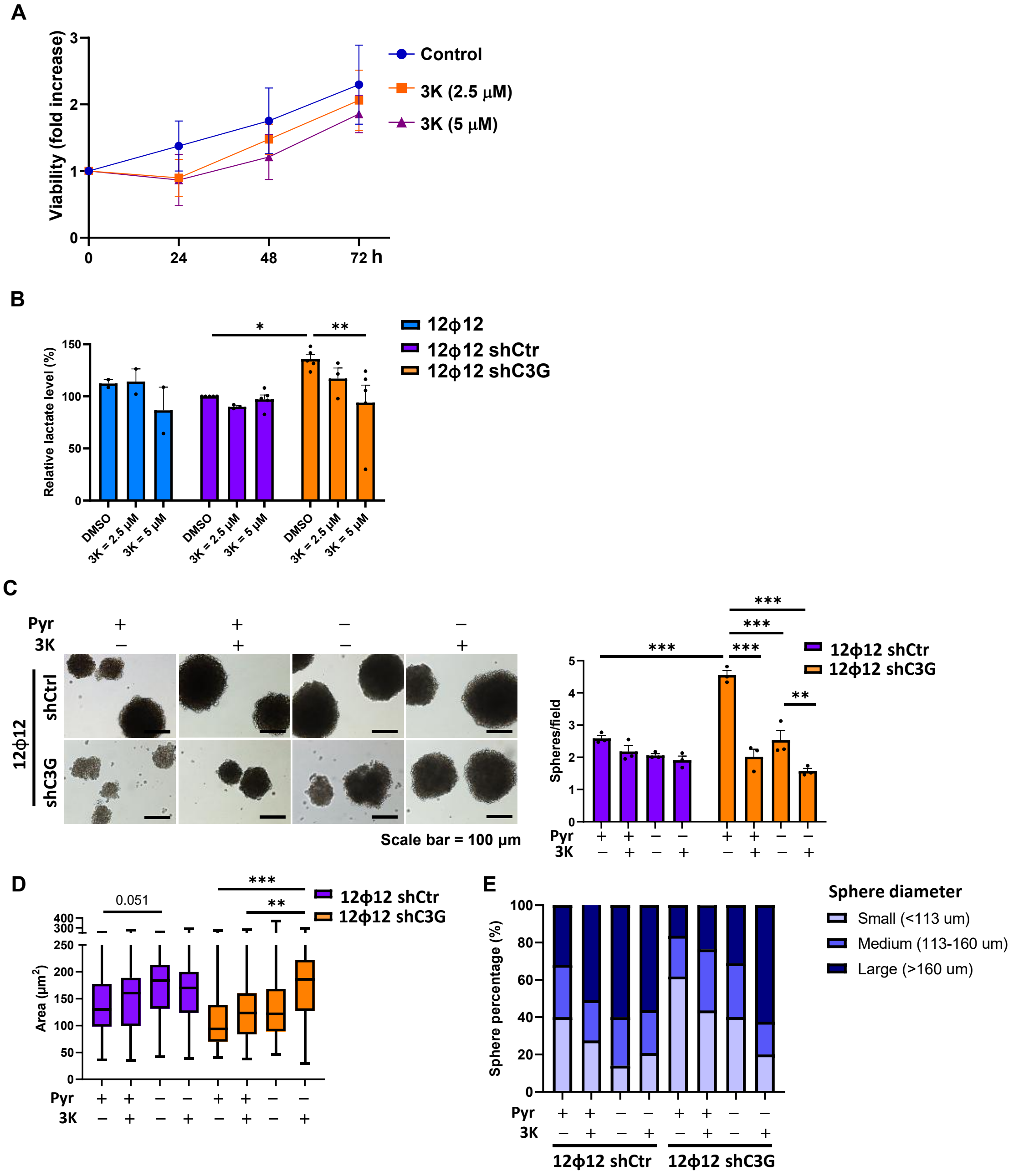

Figure S9-Effect of PKM2 inhibition with 3K inhibitor on non-tumor and GBM cells. Role of pyruvate on GBM sphere forming capacity. A) Analysis of cell viability in lung fibroblasts treated with the PKM2 inhibitor 3K (2.5 μM and 5 μM) at 24, 48 and 72h (n=3). **B)** Extracellular lactate levels in C3G-silenced (12Φ12shC3G) and non-silenced (12Φ12shControl (12Φ12shCtrl)) 12Φ12 cells treated with the PKM2 inhibitor 3K (2.5 μM

and 5 μM) (n=3). **C-E)** Effect of pyruvate on PKM2-dependent sphere-forming capacity in 12Φ12shControl and 12Φ12shC3G cells. **C)** Left panel, phase-contrast microscope images of spheres at passage 1. Right panel, histogram showing the number of spheres per field. **D)** Graphic shows the sphere sizes expressed as the area in μm^2. **E)** Graphic shows the percentage of spheres with small, medium, and large sizes. *p≤0.05, **p≤0.01 and ***p≤0.001 compared as indicated.

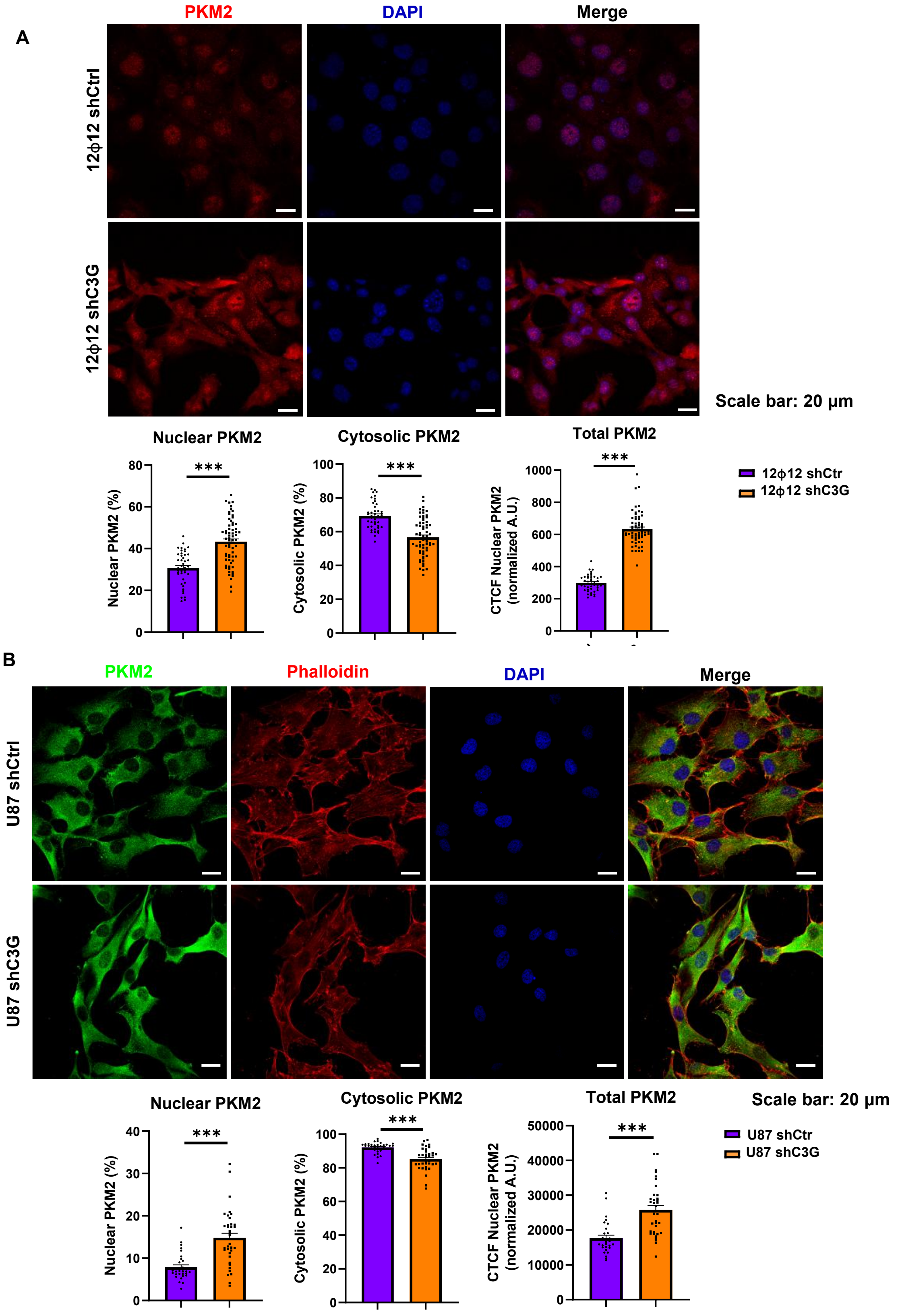

Figure S10-Effect of C3G silencing on subcellular localization of PKM2. Analysis by confocal microscopy of PKM2 subcellular localization in C3G-silenced (U87shC3G and 12Φ12shC3G) and their control cells (U87shControl (U87shCtrl) and 12Φ12shControl (12Φ12shCtrl)) cells. **A)** PKM2 (red) and nuclear DAPI (blue) staining in 12Φ12 cells. Upper panel, representative microscope images; and lower panel, quantification of

nuclear and cytosolic PKM2 staining. **B)** PKM2 (green), Rhodamine (for Actin filaments (red)) and nuclear DAPI (blue) staining in 12Φ12 cells. Upper panel, representative microscope images; and lower panel, quantification of nuclear and cytosolic PKM2 staining. ***p≤0.001 compared as indicated.

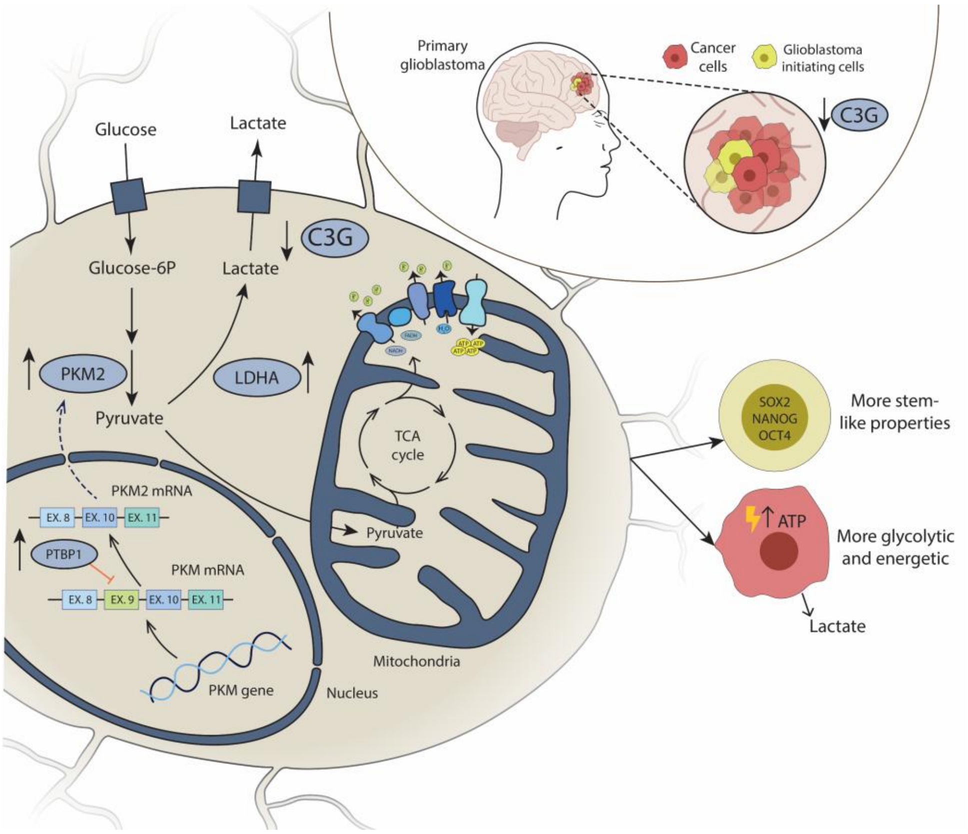

Figure S11-Graphical abstract. Scheme showing how C3G downregulation in GBM cells regulates stemness through PKM2. Low levels of C3G induces LDHA expression and promotes PKM2 expression through the upregulation of PTBP1 splicing factor, and LDHA expression, which in turn enhances glycolysis, lactate generation and stemness.
